# Kinetics of Antibody Responses and Effector Cell Sensitivity After High Dose Birch Extract Nasal Challenge

**DOI:** 10.1111/all.70365

**Published:** 2026-05-04

**Authors:** N. J. Campion, S. Villazala‐Merino, C. Morgenstern, L. Nemec, V. Stanek, A. Tu, M. Zghaebi, J. Toth, R. Fröschl, T. Perkmann, M. Bastl, K. Gangl, S. Schneider, H. Breiteneder, R. Ristl, M. Focke‐Tejkl, R. Valenta, V. Niederberger‐Leppin, J. Eckl‐Dorna

**Affiliations:** ^1^ Vienna Airway Lab, Department of Otorhinolaryngology, Head and Neck Surgery Medical University of Vienna Vienna Austria; ^2^ Department of Laboratory Medicine Medical University of Vienna Vienna Austria; ^3^ Department of Pathophysiology and Allergy Research, Center for Pathophysiology, Infectiology and Immunology Medical University of Vienna Vienna Austria; ^4^ Center for Medical Statistics, Informatics and Intelligent Systems Medical University of Vienna Vienna Austria; ^5^ Center for Molecular Allergology Karl Landsteiner University of Health Sciences Krems Austria

**Keywords:** allergic rhinitis, birch pollen allergy, late allergic response, local allergic response, nasal challenge, systemic allergic response

## Abstract

**Background:**

In allergic rhinitis (AR), effector cell activation via allergen‐specific (s) IgE cross‐linking is well established, yet the in vivo kinetics of allergen‐specific antibody responses and their modulation of effector cell reactivity after nasal allergen exposure warrant further investigation. We therefore set out to characterize the sequence and timing of systemic and local antibody responses and basophil/mast cell sensitivity after nasal allergen challenge, and their influence on subsequent seasonal responses.

**Methods:**

In this randomized, double‐blind, placebo‐controlled trial, birch pollen–allergic adults received three daily nasal challenges with birch pollen extract (*n* = 20) or placebo (*n* = 10) in autumn. sIgE, sIgG, sIgG1, sIgG4 and sIgA levels, basophil activation (BAT), and titrated skin prick test (tSPT) responses were measured biweekly in nasal mucosal lining fluid (nMLF), blood and skin for 3 months, and before, during, and after seasonal exposure.

**Results:**

Allergen but not placebo challenge induced sequential sIgE rises, first in serum, then nMLF, mirrored by sIgG responses. Basophil sensitivity peaked with serum sIgE at 4 weeks, followed by maximal mast cell sensitivity in tSPT at 8 weeks. Allergen‐specific antibodies remained elevated through the pre‐seasonal and seasonal periods. Allergen‐challenged subjects showed reduced sIgE rise and lower effector cell sensitivity during the following pollen season.

**Conclusion:**

Out‐of‐season high‐dose nasal allergen exposure enhances sIgE levels and effector cell sensitivity as well as sIgG and nasal sIgA responses. The latter appear to later attenuate seasonal amplification of allergic responses.

**Trial Registration:**

ClinicalTrials.gov ID: NCT03644680

## Graphical abstract

High‐dose birch‐extract nasal challenge induced synchronous peaks at 4 weeks in serum specific IgE (sIgE) and basophil sensitivity. Mast cell sensitivity via tSPT and sIgG1 and sIgG4 responses peaked later, at 6–8 weeks. Preseasonal allergen exposure attenuated seasonal sIgE increase and prevented further rise in tSPT sensitivity. BAS, basophil; BAT, basophil activation test, ELISA, enzyme‐linked immunosorbent assay; MC, mast cell; NC, nasal challenge; sIg, specific immunoglobulin; tSPT, titrated skin prick test; w, weeks.

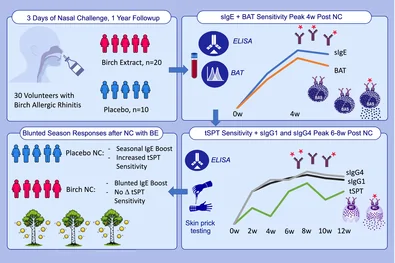

**Abbreviations**
AR: allergic rhinitis
BAT: basophil activation test
BPE: birch pollen extract
EAR: early allergic/phase response
Ig: immunoglobulin
IL: interleukin
IL‐13^NR^
: IL‐13 non‐responder
IL‐13^R^
: IL‐13 responder
LAR: late allergic/phase response
Log2FC: Log2 fold changes
NC: nasal challenge
nMLF: nasal mucosal lining fluid
PNIF: peak nasal inspiratory flow
(s): allergen specific
SPT: skin prick test
TNSS: Total Nasal Symptom Score
VAS: Visual Analogue Scale

## 1 Introduction

Allergic rhinitis (AR) is a major inflammatory disease of the nose and is characterised by sneezing, rhinorrhoea, nasal obstruction and nasal itch [1]. It affects 20%–30% of people in Western countries [2, 3] and causes substantial economic burden, 3.4 billion USD annually in the USA and 3.5–4 billion Euros in Europe [4, 5]. Treatments include allergen avoidance, symptom management, and allergen immunotherapy, which is the only disease‐altering therapy [2, 6, 7].

IgE binds its high‐affinity receptor (FcεRI) on mast cells and basophils, mediating effector cell activation and immediate allergic symptoms. Upon allergen re‐exposure, cross‐linking of receptor‐bound allergen‐specific (s) Ig triggers rapid mediator release driving acute allergic inflammation [2]. In addition, the allergen crosslinks IgE bound to FcεRI on dendritic cells as well as the low affinity receptor CD23 mainly on B cells and monocytes thereby promoting IgE‐facilitated antigen presentation, an essential process governing mucosal T‐cell reactivation during the effector phase of allergic disease [8, 9]. Re‐exposure to the culprit allergen leads to rises in circulating sIgE peaking at around 4–6 weeks after nasal allergen contact [10, 11]. Although sIgE correlates with FcεRI expression and mast cell mediator release in vitro, the in vivo kinetics of effector cell sensitivity changes following allergen exposure are an important area of research [12]. Available data, mainly from IgE depletion by immune apheresis or neutralisation with omalizumab, suggest that complete removal of IgE requires 4–12 weeks to downregulate basophil and mast cell sensitivity [13, 14]. Far less is known about whether allergen‐induced increases in systemic sIgE enhance mast cell and basophil sensitivity. Direct comparisons of nasal challenge (NC) with allergen versus natural allergen exposure on systemic antibody responses and effector cell sensitivity are lacking. Furthermore, data on the kinetics of allergen‐specific IgG, IgG1, IgG4 and IgA in serum and nasal secretions after allergen exposure in allergic individuals are limited. Characterising these responses may clarify their relationship to IgE‐driven effector cell activation [15]. Furthermore, work from our group and others have shown that significant IL‐13 responses in the late phase of allergic responses after NC with allergen distinguishes patients who suffer from pronounced clinical late phase reactions [16, 17, 18]. Whether this then effects systemic allergic immune antibody responses in the weeks after NC with allergen remains unexplored.

In this randomised, placebo‐controlled, double‐blind prospective study, we examined the timing of mast cell and basophil sensitivity changes and correlated these with serum and nasal sIgE levels in birch pollen‐allergic volunteers challenged nasally with birch pollen extract or saline in autumn outside the birch pollen season. Participants were then followed until the following birch pollen season in spring to track these changes and assess the impact of pre‐seasonal allergen exposure on seasonal allergic responses.

## 2 Methods

### 2.1 Study Design and Controlled Nasal Challenge

Ethical approval for this double‐blind placebo‐controlled nasal provocation study was obtained from the ethical committee of the Medical University of Vienna (EK No. 1253/2018). The study was powered to detect changes in allergen‐specific IgE responses and how they related to changes in mast cell (titrated skin prick test) and basophil sensitivity (basophil activation test). All other analyses are considered exploratory. Prior to study inclusion, written informed consent was obtained from each volunteer according to the Declaration of Helsinki. Out of 52 birch pollen allergic volunteers screened, 30 adult Caucasian participants with confirmed birch pollen allergy were included. Only participants meeting all entry and exclusion criteria (detailed list: clinicaltrials.gov ID: NCT03644680) were included. To be defined as having birch induced allergic rhinitis, volunteers had to fulfil all of the following criteria: a positive skin prick test (SPT) to birch pollen extract, allergen‐specific IgE levels to Bet v 1 above alternate scoring method (ASM) class 3 (> 1.4 kUA/L) in the ImmunoCAP and reported allergic rhinitis symptoms over at least two consecutive Austrian birch pollen seasons requiring the use of symptomatic medication. For details regarding their co‐sensitization and co‐occurrence of asthma and conjunctivitis, see Table S1 and our previous publication in this cohort [16]. All recruited volunteers lived in the federal state of Vienna and were thus all within 20 km of the city. Therefore, they received the same exposure as the source area of a pollen monitoring station is approximately 30 km [19] and the pollen monitoring trap is located in the centre of Vienna, hosted by the pollen service Austria.

Prior to nasal challenge (NC) volunteers attended a baseline visit (N0), where a detailed medical history as well as blood (heparinized, serum) and nasal samples (nasal mucosal lining fluid = nMLF) were obtained and a titrated SPT (tSPT) for birch was performed (Figure 1A).

**FIGURE 1:**
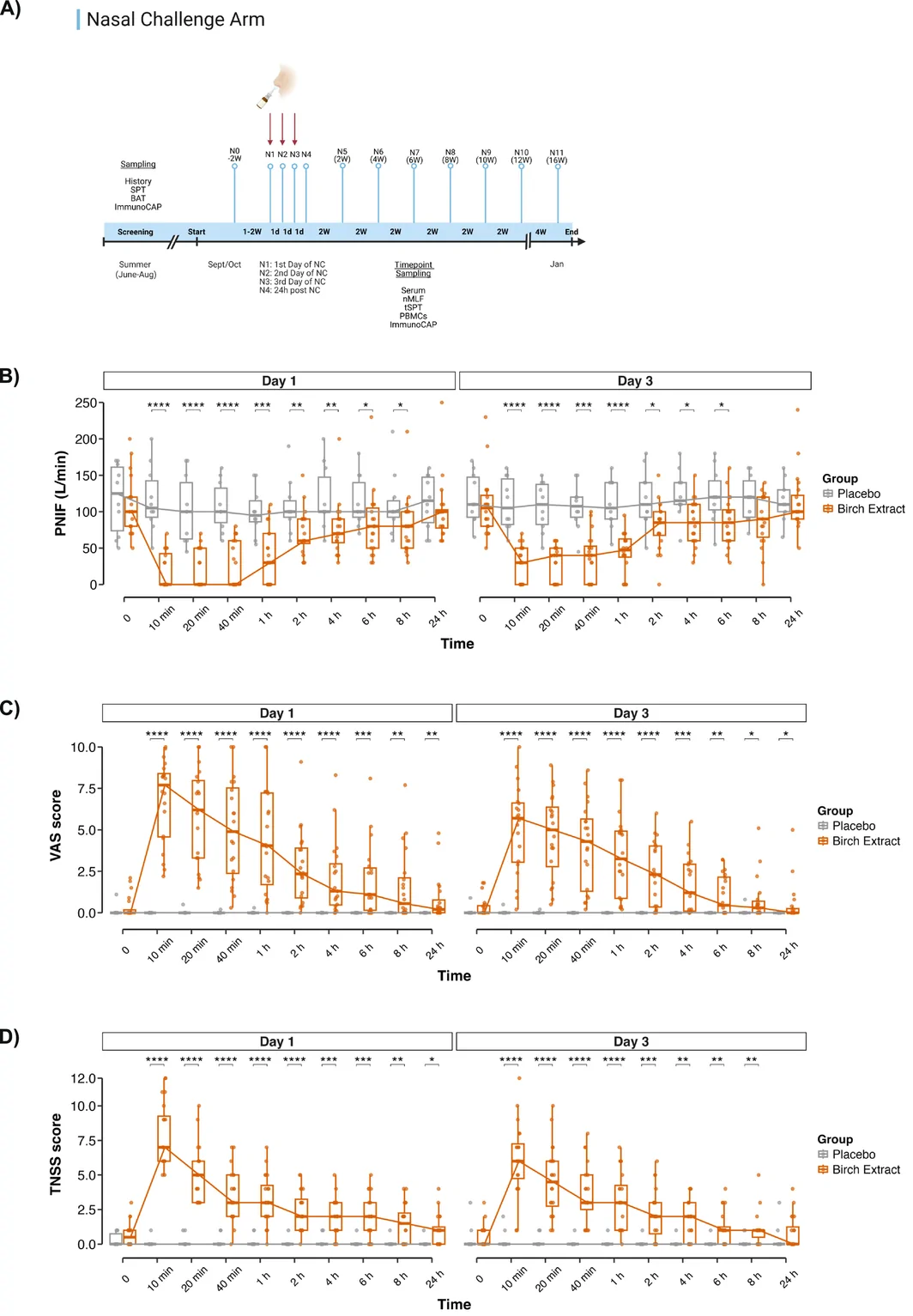
Overview of study design and clinical response on Days 1 and 3 of NC with allergen. (A) Birch pollen allergic subjects were screened in summer after the birch pollen season and those meeting the inclusion criteria attended a baseline visit (N0) taking place at a maximum of 2 weeks before the start of the study (autumn of the same year). On three consecutive days at N1, N2 and N3, volunteers underwent nasal challenge (NC) with either birch pollen extract or 0.9% saline solution (placebo). Thereafter volunteers were sampled every 2 weeks for 12 weeks (N5‐N10) with a final visit 16 weeks after NC with allergen (N11) (January). (B–D) Boxplots show clinical response to nasal provocation with birch pollen extract BPE (orange, *n* = 20) or placebo (grey, *n* = 10) during provocation Days 1 and 3 over the sampling time course (*x*‐axis) in the peak nasal inspiratory flow (PNIF) (*y*‐axis, Litres of airflow per min) (B), in visual analogue scale (VAS) score (*y*‐axis, cm) (C) and in total nasal symptom score (TNSS) score (*y*‐axis, mean score, max possible = 12) (D). Boxplots show distribution of values with median (horizontal line within box), quartiles (upper and lower box boundaries) and whiskers extending to 1.5 times the interquartile range. Lines connect the median values across time. BAT, basophil activation test; BPE, birch pollen extract; NC, nasal challenge; nMLF, nasal mucosal lining fluid; PBMCs, peripheral blood mononuclear cells; tSPT, titrated skin prick test. Asterisks represent statistically significant differences between groups (*p ≤ 0.05, **p ≤ 0.01, ***p ≤ 0.001, ****p ≤ 0.0001). Figure 1A created in https://BioRender.com.

Volunteers were stratified according to their baseline Bet v 1 specific IgE levels and randomised in a 2:1 ratio to receive nasal challenge with placebo (0.9% sodium chloride) or Birch Pollen Extract (Allergopharma, Reinbeck, Germany) (BPE; *n* = 20, placebo; *n* = 10). This randomisation was powered to be able to detect significant Ig responses in allergen provoked as opposed to placebo provoked volunteers. To avoid volunteers having simultaneous environmental exposure of pollen from birch trees (March–May) or trees containing cross‐reactive allergens (January–February), the controlled nasal provocation was performed on three consecutive days far outside of the pollinating seasons (October). At the time of provocation all patients confirmed they had no active allergic symptoms. The dose of allergen for Birch Pollen Extract (BPE)‐challenged group this accounted for 20 μg/mL of Bet v 1 in each nostril per provocation. Bet v 1 concentration was determined by ELISA using purified Bet v 1 as standard as previously described [20]. The NC procedure was performed in compliance with the EAACI position paper [21]. In brief, challenges were performed in a quiet room with standardised conditions (20°C, 50% humidity) after allowing 15 min of acclimatisation. Challenge was performed using the same lot of a standardised birch pollen extract throughout the study using the Unidose Aptar nasal spray devices (AptarGroup Inc., Crystal Lake, Illinois, United States) delivering 100 μL per puff and nostril. Challenge solutions were prepared the same day of nasal provocation in glass vials at a max of 2 h before use. All BPE challenged patients showed an objective (PNIF decrease > 40%) and subjective (TNSS rise > 5 points) positive reaction.

After 3 days of consecutive NC (N1‐N3) with birch pollen extract (BPE) or placebo, volunteers were followed up every 2 weeks until 3 months post NC (N5‐N10) with a final visit at 4 months (N11) (Figure 1A). On days N1 and N3, volunteers underwent a 24‐h time course (0, 10, 20, 40, 60 min; 2, 3, 6, 8 h) where peak nasal inspiratory flow (PNIF), Total nasal symptom score (TNSS), and symptoms evaluated by visual analogue scale (VAS) were recorded. At visits N5‐N11, tSPT was performed and serum and nMLF samples were taken.

During a second observation period, the same study volunteers underwent 4 further visits at 4 weeks before the birch season (S1), and then at 4 (S2), 8 (S3) and 20 weeks after the season (S4) (Figure S1). Volunteers were asked to keep a symptom medication diary throughout the season which they were asked to fill out at the end of each day and were sent weekly reminders to try and reduce potential recall bias.

For more detailed descriptions of the methodology used see Supporting Information.

### 2.2 Nasal Sampling

Nasosorptions (Nasosorption FX‐I, Hunt Developments (UK) Limited, Midhurst, West Sussex, United Kingdom) were used for the collection of nMLF and were processed as previously described [22].

### 2.3 SPT at Screening Visit

All volunteers were screened with a panel of 15 inhalative allergen extracts, including birch pollen using a commercial skin prick test set from Bencard Allergie (Munich, Germany).

### 2.4 tSPT of Birch Pollen Extract

We performed a titrated skin prick test, using a commercially available birch pollen skin prick test solution (Bencard Allergie, 50,000 specific bioequivalent units (SBE)/mL). Undiluted extract was the starting point and this was then diluted further at 1/3 steps down to a concentration of 1 SBE/mL (11 total dilutions). All solutions, including histamine as a positive control, were applied in quadruplicates in a randomized manner. Endpoint titrations (the lowest concentration a volunteer had a positive reaction to) were recorded for each visit. The titrated skin prick test (tSPT) results were analysed by converting endpoint titrations into logarithmic changes in sensitivity relative to baseline. For example, if a volunteer's endpoint titration to birch extract increased from dilution 6 at baseline to dilution 7 at follow‐up, this was recorded as a log change or reactivity score of +1, which in real terms corresponds to a threefold increase in sensitivity due to the 1/3 dilution steps used. Conversely, a decrease in sensitivity to dilution 5 would correspond to a reactivity score of −1 or a threefold decrease in sensitivity. Log changes in sensitivity were plotted over time for each participant. For further information see Supporting Information.

### 2.5 Basophil Activation Test (BAT)

The basophil activation (BAT) was performed as previously described to determine basophil sensitivity to the allergen [23, 24] under standardised pre‐analytical conditions (morning sampling, processing within 2 h, antihistamine washout ≥ 3 days) to minimise biological and technical variability. Briefly, stimulation cocktails consisting of differing concentrations of recombinant (r)Bet v 1 (Biomay, Vienna, Austria; Isoform Bet v 1a, Bet v 1.0101 (starting concentration: 10 ng/mL, then 1/3 dilution steps until 0.004 ng/mL as indicated in the respective figures)), N‐Formylmethionyl‐leucyl‐phenylalanine (fMLP) (Positive Control, 12 μM (Abcam, Cambridge, United Kingdom)), monoclonal human‐anti‐IgE (Positive Control, final concentration 1 μg/mL (Beckham Coulter, Vienna, Austria)) or buffer only (Negative control, HEPES Buffer) were prepared in triplicates in HEPES Buffer (20 mM HEPES, 133 mM NaCl, 5 mM KCl, 7 mM CaCl_2_, 1 mg/mL BSA, pH 7.4). Aliquots of 100 μL of heparinised blood were then mixed with 20 μL of the stimulation cocktails within 3 h of blood draw and incubated at 37°C, 5% CO_2_ for 15 min in a 96 deep well plate (Starlab, Milton Keynes, United Kingdom). The reaction was then stopped by adding 10 μL of STOP buffer (20 mM HEPES, 133 mM NaCl, 5 mM KCl, 20 mM EDTA, pH 7.4) to each well. Afterwards 20 μL of the antibody staining mixes (detailed below) diluted in staining buffer (1× PBS, 0.1% BSA) were added to each well and the plates were left to incubate in the dark at room temperature for 20 min. The following antibodies were used: anti‐human CD193 FITC (Biolegend, San Diego, USA), anti‐human CD63 PE (Biolegend, San Diego, USA), eFluor780 viability dye (Thermo Fisher Scientific, Waltham, USA) and anti‐human CD123 BV421 (Biolegend, San Diego, USA). Thereafter, 800 μL of 1× 1‐step Fix/Lyse solution (Invitrogen eBioscience, Thermo Fisher Scientific, Waltham, USA) was added to each well and left to incubate for 10 min in the dark. The samples were then centrifuged (1000 rpm for 5 min), then washed with 800 μL of staining buffer before being spun down once more. Samples were subsequently taken up in 300 μL of staining buffer before being analysed on FACS Canto II cytometer (BD Biosciences, New Jersey, USA). Basophils were identified as alive cells staining positively for CD193 and CD123 and samples were acquired until at least 500 basophils were present per sample and analysed with FlowJo Software (version 10; FlowJo, Ashland, Oregon, USA). The percentage of CD63 positive cells was determined at each concentration and the percentage of CD63 positive cells present in unstimulated samples were subtracted as background. For each individual patient and timepoint, the percentage of CD63 positive cells for all concentrations was calculated and depicted in a curve. The drm function from the drc (v3.0‐1) R package [25] was used to fit a 4‐parameter log‐logistic dose–response model to the data at each time point and for each group. The parameters estimated were the slope, the lower and the upper limits and the median effective dose (ED50), median was calculated and is shown in the respective graphs.

### 2.6 Serum and nMLF Ig Measurements

Levels of Bet v 1 specific IgE, IgG, IgG1, IgG4 and IgA in serum and nMLF were measured by ELISA by coating the plates with recombinant (r)Bet v 1 at 1 μg/mL (Biomay, Vienna, Austria; Isoform Bet v 1a, Bet v 1.0101). We also measured total and Bet v 1 specific IgE levels in serum samples by ImmunoCAP (Thermo Fisher, Uppsala, Sweden) as per manufacturer's instructions. To ensure the changes in Ig levels could be compared across patients, fold change from baseline was calculated for each patient with the following formula: Fold Change in Ig = Optical Density of Visit/Optical Density of Baseline or, in the case of IgE and IgG4 where standards were available, Fold Change in Ig = Concentration of Visit/Concentration of Baseline. The ELISA was designed and used as a semi‐quantitative assay, and measurements were expressed as optical density (OD) values and log2 fold‐change (log2FC) relative to controls. As an example, a log2FC of 1.06 = 2.06 fold change in Ig levels from baseline. For detailed information, see Supporting Information.

### 2.7 Measurement of Clinical Parameters and Seasonal Diary

On Day 1 and Day 3 of NC (N1 and N3), the following parameters were assessed at baseline and then at 10, 20, 40, 60 min as well as 2, 4, 6, 8, and 24 h post NC:

1. Volunteer's nasal air flow using an in‐check portable nasal inspiratory flow (PNIF) metre (Clement Clarke International, Harlow, United Kingdom) [26].
2. Modified Total Nasal Symptom Score (TNSS) [27].
3. A visual analogue scale (VAS) 10 cm line was used to assess overall allergic symptoms at each time point (0 indicated no symptoms and 10 indicated severe symptoms that are hard to tolerate) [16].

During the birch pollen season, volunteers were asked to keep a paper diary of their daily combined symptom medication scores, which was used and calculated as previously described [28].

### 2.8 Definition of Early and Late Clinical Allergic Responses

In the early phase of the allergic response (< 60 min after provocation) a positive reaction was defined clinically as a rise in the VAS to 5 cm or above and a drop in the PNIF by at least 40%. With regards to the late phase (2–8 h after provocation) this was defined clinically based on the works of Kim et al. [29] and Soliman and Ellis [30] as a 25% or more decrease in their nasal airflow as measured by PNIF from baseline levels and an increase in the nasal obstruction symptom parameter of the TNSS compared to baseline at 6 h post provocation.

### 2.9 Environmental Pollen Concentrations

The airborne pollen concentrations (pollen grains/m^3^ air) were evaluated daily during the birch pollen season as previously described [31].

### 2.10 Definition of IL‐13 Responder Status

Based on previously published data in this cohort, a secondary post hoc analysis looking into IL‐13 responders (IL‐13^R^) and non‐responders (IL‐13^NR^) was performed as an additional exploratory analysis and these subgroups were defined as previously described [16]. Briefly, IL‐13^R^ were defined as those having increases in IL‐13 in nMLF above the 90th percentile of the placebo group during the LAR (2–24 h) at visit N1 at one or more time point(s).

### 2.11 Data and Statistical Analysis

Statistical analysis of the collected data was performed using R version 4.4.1 (R Foundation for Statistical Computing, Vienna, Austria) in RStudio (v2024.09.0+375). For a detailed description, see Supporting Information. Analyses were conducted using all available participant data without exclusion for missing follow‐up. Three participants in the BPE group discontinued the study (one after Week 6 and two after Week 16), and two additional participants missed one scheduled visit but otherwise remained enrolled. Missing data were not imputed, and participants contributed observations until discontinuation or missed visits.

## 3 Results

### 3.1 Reduced Nasal Inspiratory Flow and Distinct Nasal Symptoms Following NC With BPE

At the baseline visit (N0, Figure 1A), birch pollen allergic volunteers (for baseline characteristics of the volunteers and baseline Ig Isotype levels please refer to Tables S1 and S2) were randomly allocated to receive NC with BPE (*n* = 20) or placebo (*n* = 10) over three consecutive days (N1–N3). Thereafter, volunteers were sampled biweekly for 12 weeks, with a final visit at 16 weeks after NC (N4–N11). Three volunteers dropped out of the birch challenge arm during the study. One dropped out after week 10 (visit N9), and the other two volunteers dropped out after week 16 (N11) (Figure S1).

To assess clinical response to NC, changes in airflow (PNIF) and symptom burden (TNSS and VAS) on Day 1 and Day 3 were measured. Following nasal provocation, the median PNIF value was significantly diminished 10 min after NC in the BPE compared to the placebo group and remained reduced up to 8 h post‐challenge on Day 1 and 6 h post‐challenge on Day 3 (average *p* = 0.0098) (Figure 1B). Subjects challenged with BPE also reported a significantly higher symptom burden in VAS (average *p* = 0.0043, Figure 1C) and TNSS (average *p* = 0.0021, Figure 1D) than placebo challenged subjects.

### 3.2 Nasal Allergen Contact Leads to Rises in Bet v 1 Specific IgE in Serum and nMLF 4 Weeks After NC With Allergen

Serum and nasal Bet v 1 sIgE rose at 2 weeks and the peak responses were seen at 4 weeks post NC (median log2 fold change at 4 weeks (Log2FC = 1.06 (IQR = 0.84); *p* = 4.66 × 10^−7^; Log2FC = 1.27 (IQR = 1.95); *p* = 2.44 × 10^−2^ for serum and nMLF, respectively; Figure 2A,B, Figure S2)).

**FIGURE 2:**
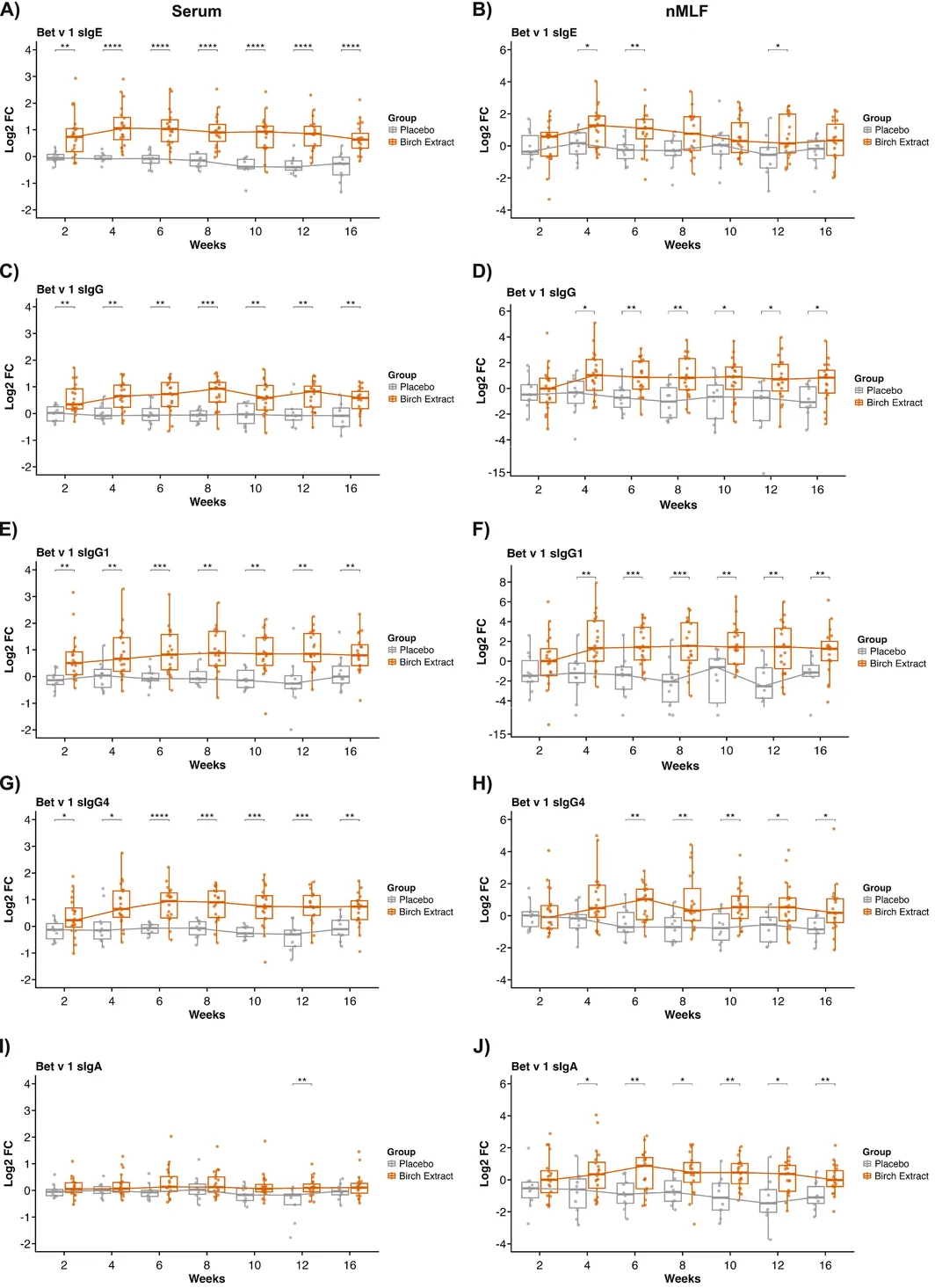
Changes in allergen‐specific immunoglobulin levels to nasal birch pollen extract (BPE, orange, *n* = 20) or placebo (grey, *n* = 10) challenge in serum and nMLF over time. Left panel boxplots (A, C, E, G, I) show log2 fold changes compared to baseline (*y*‐axis) of Bet v 1 specific IgE (A), IgG (C), IgG1 (E), IgG4 (G), and IgA (I), across time (*x*‐axis, weeks) in serum measured by ELISA. Right panel boxplots (B, D, F, H, J) show log2 fold changes compared to baseline (*y*‐axis) of Bet v 1 specific IgE (B), IgA (D), IgG (F), IgG1 (H), and IgG4 (J) across time (*x*‐axis, weeks) in nMLF measured by ELISA. Boxplots show distribution of values with median (horizontal line within box), quartiles (upper and lower box boundaries), and whiskers extending to 1.5 times the interquartile range. Lines connect the median values across time. Asterisks represent statistically significant differences between groups (*p ≤ 0.05, **p ≤ 0.01, ***p ≤ 0.001, ****p ≤ 0.0001). Log2FC = log2 fold change; nMLF = nasal mucosal lining fluid.

### 3.3 Nasal Challenge With Allergen Induces Nasal, but Not Systemic, Bet v 1‐Specific IgA and Elicits an Early sIgG1 Peak Followed by sIgG4 Both in Serum and nMLF

The rise in sIgE (Figure 2A,B) was accompanied by a rise in sIgG (median Log2FC = 0.93 (IQR = 0.74); *p* = 1.9 × 10^−4^; Log2FC = 1.02 (IQR = 2.40); *p* = 3.92 × 10^−2^, for serum and nMLF, respectively; Figure 2C,D) including both IgG1 and IgG4 subclasses (Figure 2E–H), which peaked at 6–8 weeks in serum and at Week 4 in nMLF. For sIgA, significant changes were detected starting at 4 weeks after allergen provocation in the nose, but not the serum (Figure 2I,J). No relevant changes in serum or nasal Ig levels were observed following NC with placebo (Figure 2A–J).

### 3.4 Increases in Basophil and Mast Cell Sensitivity Follow Rises in Allergen Specific IgE

Basophil activation tests (BAT) were performed as an in vitro parameter of basophil sensitivity and titrated skin prick test (tSPT) as an in vivo parameter to assess mast cell sensitivity. In the BAT, we observed increased sensitivity at Week 4 in the BPE‐provoked subjects, which was significantly different to placebo at a concentration of 0.370 ng/mL of Bet v 1. (Figure 3A, median percent activation: BPE: 57.37% (IQR = 39.38%) and Placebo: 14.13% (IQR = 44.34%); *p* = 0.044) in line with the peak of sIgE in the serum (Figure 2A). Furthermore ImmunoCAP sIgE in Serum correlated positively with CD63% upregulation at 4 weeks (Figure S3A) and in a lead lag analysis serum sIgE levels at 2 weeks were positively correlated with CD63% upregulation in the BAT at 4 weeks post NC (Figure S3C). No significant correlations were found with sIgE in nMLF (Figure S3B,D). Basophil sensitivity remained elevated in BPE as compared to placebo challenged subjects although significance was lost. Furthermore basophil reactivity was significantly elevated in the BPE‐provoked subjects as compared to placebo provoked from Week 8 onwards (Figure 3B, Table S3). A significant difference in mast cell sensitivity between the BPE and placebo group occurred later and was detectable 6 weeks after NC and peaked at 8 weeks (median reactivity score = 1.5, median absolute deviation (MAD) = 0.74; *p* = 2.9 × 10^−3^) (Figure 3C).

**FIGURE 3:**
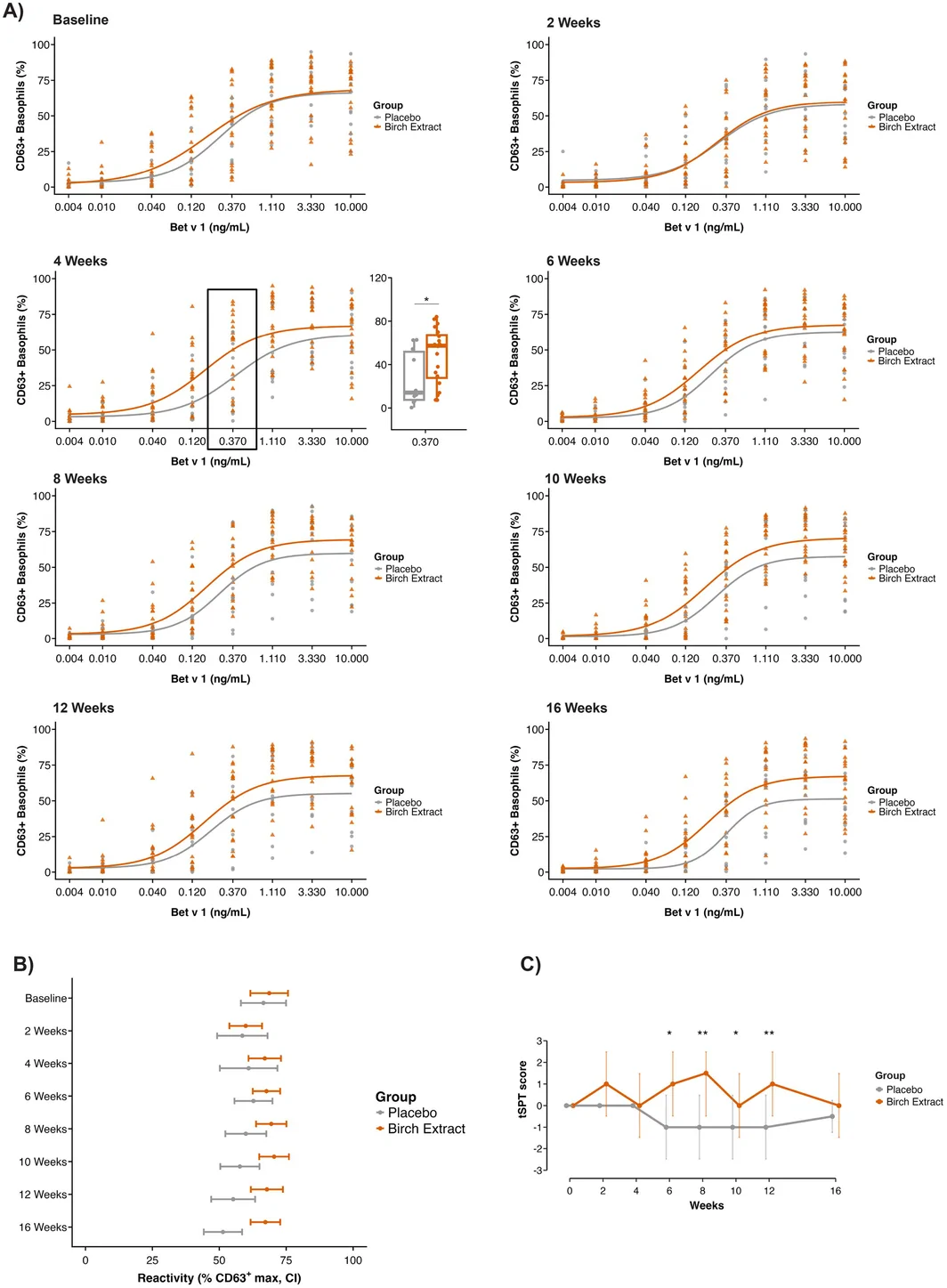
Changes in basophil activation test (BAT) and titrated skin prick test (tSPT) sensitivity during 16 weeks following nasal challenge with birch pollen extract (BPE, orange, *n* = 20) or placebo (grey, *n* = 10). Changes in BAT sensitivity (A) shown via fitted dose–response curves at each time point over 16 weeks following nasal challenge (*x*‐axis, concentration of Bet v 1 in ng/mL; *y*‐axis, percentage (%) of total basophils positive for CD63). Boxplot at 4 weeks illustrates the median response at the highlighted concentration of BPE vs. placebo provoked volunteers with whiskers extending 1.5 times the interquartile range and individual volunteers as points on top. (B) Forest plot of BAT reactivity at each time point over 16 weeks following nasal challenge (*x*‐axis, maximum CD63% upregulation with confidence intervals, *y*‐axis, time in weeks). (C) Median log change from baseline in endpoint titration in the birch extract tSPT. Asterisks represent statistically significant differences between groups (*p ≤ 0.05, **p ≤ 0.01, ***p ≤ 0.001, ****p ≤ 0.0001). CD, cluster of differentiation; tSPT, titrated skin prick test.

### 3.5 Volunteers Challenged With BPE Exhibit a Blunted Boost of IgE Production and Effector Cell Sensitivity Following Seasonal Allergen Exposure

To obtain mechanistic insight into how pre‐seasonal allergen challenge shapes Ig responses and effector cell sensitivity during the subsequent season, we evaluated immunoglobulin levels and effector cell responsiveness during in‐season exposure (Figure 4A–H). The study was conducted during the 2019 birch pollen season which ranged from March 30th (day of year (DOY) 89) to May 2nd (DOY 122), with a peak period from March 31st (DOY 90) to April 22nd (DOY 112), following the clinical trial season definition [32]. The annual pollen integral (APIn) was 11,773, nearly twice the 50‐year average in Vienna (5883 [33]) and hence signals an intense pollen season. Though volunteers without pre‐seasonal allergen exposure showed significantly higher increases in serum Bet v 1 sIgE in ImmunoCAP compared to pre‐seasonal BPE‐provoked volunteers starting at 4 weeks after the birch pollen season (median Log2FC = 0.47 (IQR = 0.48); *p* = 8.38 × 10^−3^) (Figure 4C), we could not observe any difference between the groups in the combined symptom medication score (CSMS) throughout the season (Figure 4B). Bet v 1‐specific IgE levels in nMLF in pre‐seasonal BPE‐provoked and volunteers without pre‐seasonal allergen exposure remained unaltered and there were no differences between the groups (Figure 4D). Bet v 1‐specific IgG, IgG1, IgG4 or IgA levels remain unaltered in the volunteers without pre‐seasonal allergen exposure in serum (Figure 4E, Figure S4). A decline of Bet v 1‐specific IgG in nMLF (Figure 4F) and of IgG1 and IgG4 in serum and nMLF in BPE‐provoked group was noted (Figure S4A–D). Interestingly, Bet v 1‐specific IgA levels dropped only in the nMLF of BPE‐provoked patients but not in serum (Figure S4E,F). We did not observe a significant difference in basophil sensitivity before and after the birch pollen season (Figure 4G and Figure S4G). After the birch pollen season, birch pollen‐specific skin sensitivity was significantly lower in patients who received pre‐seasonal BPE provocation compared with those without pre‐seasonal allergen exposure, suggesting the tSPT as a potential biomarker that still requires further validation (Figure 4H).

**FIGURE 4:**
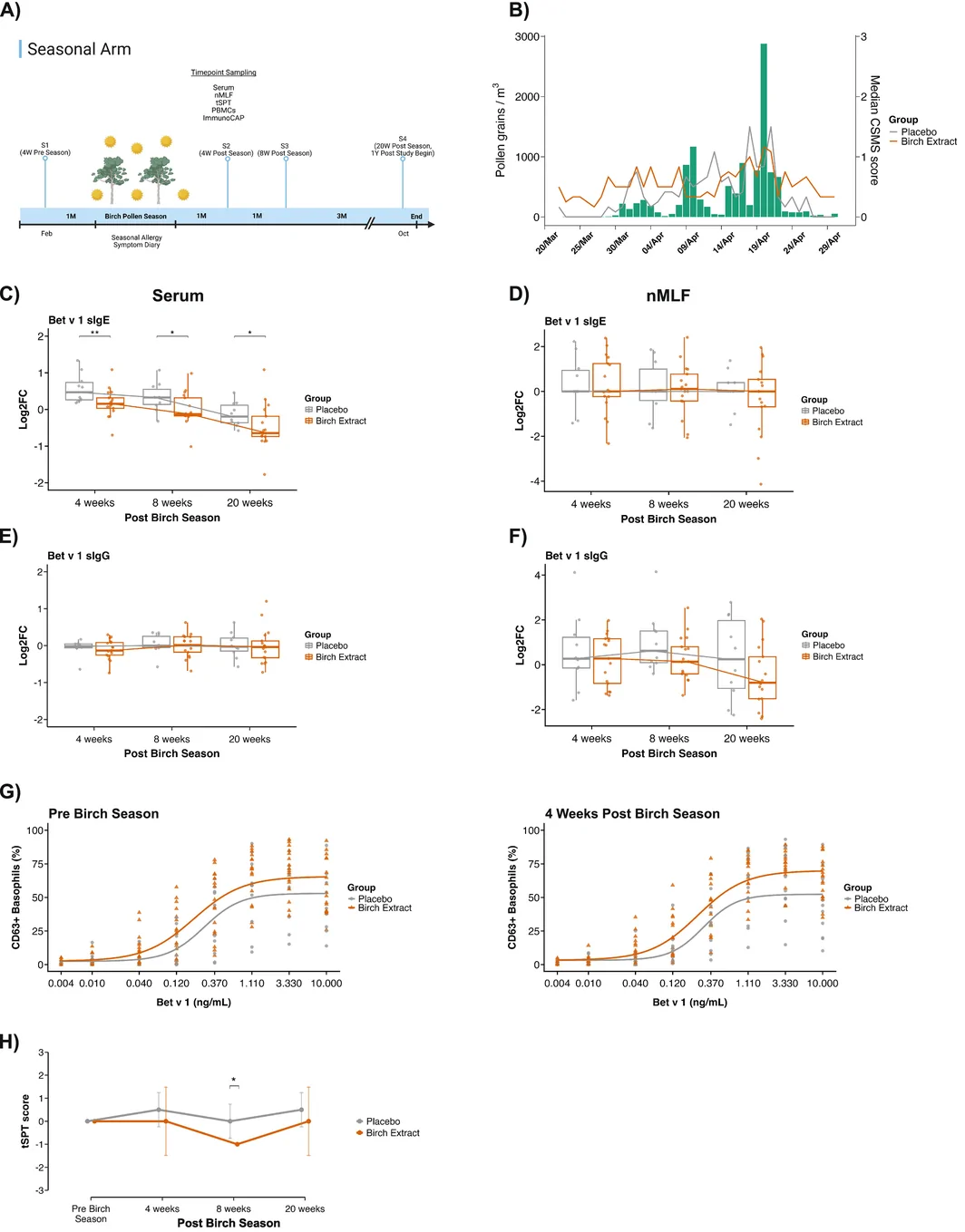
Overview and selected changes in immune and clinical parameters in the seasonal arm of the study in subjects provoked with nasal birch pollen extract (BPE, orange, *n* = 20) or placebo (grey, *n* = 10). (A) Diagram displaying an overview of the seasonal arm of the study which consisted of 4 visits (S1–S4), created in https://BioRender.com. (B) Overlay of combined median symptom medication scores (right *y*‐axis, score 0–3) and birch pollen concentrations (left *y*‐axis, pollen grains/m^3^) against time (*x*‐axis, days). (C–F) Log2FC fold changes compared to baseline (*y*‐axis) of Bet v 1 specific IgE and IgG across time (*x*‐axis, weeks) in serum (IgE (C), IgG (E)) and nMLF (IgE (D), IgG (F)) measured by ImmunoCAP (C) and ELISA (D–F). (G) Changes in BAT sensitivity shown via fitted dose–response curves pre Birch season (left panel) and 4 weeks post season (right panel) (*x*‐axis, concentration of Bet v 1 exposure in ng/mL, *y*‐axis, % of total basophils positive for CD63). (H) Changes in endpoint titration as compared to baseline in the birch extract tSPT in volunteers provoked with BPE (orange) and placebo (grey). Boxes represent interquartile range (IQR) with whiskers extending 1.5 times the IQR. Lines are connecting the medians across time. For (H) points represent the median and for (C–G) points represent individual volunteers. Asterisks represent statistically significant differences between groups (*p ≤ 0.05, **p ≤ 0.01, ***p ≤ 0.001, ****p ≤ 0.0001). BAT, basophil activation test; CD, cluster of differentiation; CSMS, combined symptom medication score; Ig, immunoglobulin; nMLF, nasal mucosal lining fluid; PBMCs, peripheral blood mononuclear cells; tSPT, titrated skin prick test.

### 3.6 Volunteers With Late Allergic Responses (LAR) Showed Increased Allergen Specific IgE Levels in Serum but Not in nMLF After Allergen Contact

In a previous analysis of this cohort, volunteers were grouped by nasal IL‐13 responses on Days 1 and 3 after provocation. Eight BPE‐challenged subjects were IL‐13 responders (IL‐13R), showing strong late allergic responses (LAR) within 8 h, whereas 12 were IL‐13 non‐responders (IL‐13NR) without LAR [16]. We were therefore interested to investigate the effect of a pronounced LAR on systemic IgE levels and effector cell sensitivity in the weeks after provocation. To this end we stratified the volunteers according to their IL‐13 responder status (Figure S6A,B); we found that IL‐13^R^ had a trend for greater Bet v 1 serum sIgE increase, which became significant at 8–12 weeks post NC to both placebo and IL‐13^NR^ in serum but only against placebo in nMLF (median Log2FC = 1.56 (IQR = 1.41); *p* = 2.74 × 10^−4^) (Figure S5A and Figure S7A). Similar though less pronounced patterns were observed for sIgG1 and sIgG4 (Figure S5B,C). There was no difference in sIgG1 and sIgG4 between IL‐13^R^ and IL‐13^NR^ in nasal nMLFs (Figure S7B,C). Regarding effector cell sensitivity, we saw a trend for greater sensitivity to Bet v 1 in the BAT in the IL‐13 responder group at baseline and at week 6 and 8 (Figure S5D), but this trend was not sustained over time. In the tSPT, no consistent significant differences between IL‐13^R^ and IL‐13^NR^ were observed (Figure 5SE). During seasonal exposure IL‐13^NR^ appeared to have a lower CSMS score, but there were no significant differences in Ig levels or effector cell sensitivity observed between these two groups during seasonal allergen exposure (Figure S8A–G). We only noted that the drop of Bet v 1‐specific serum and nMLF was more pronounced in the IL‐13^R^ than in the IL‐13^NR^ at 20 weeks (Figure S8B,C). Bet v 1‐specific IgG decreased in the nMLF in the IL‐13^R^ and in the IL‐13^NR^ but not in the placebo group (Figure S8E).

## 4 Discussion

In this study, we characterised the kinetics of sIg and effector cell sensitivity following controlled nasal allergen exposure using biweekly sampling over 3 months. A key strength of the study was preseasonal high‐dose exposure with follow‐up during the subsequent pollen season, including a placebo control group to isolate effects of natural pollen exposure. Four weeks after nasal allergen challenge, nasal and serum Bet v 1 sIgE peaked in BPE but not placebo‐treated volunteers and were strongly correlated. sIgG1 and sIgG4 showed similar but delayed peaks, and seasonal pollen exposure did not further alter Ig responses. The sIgE peak coincided with maximal basophil sensitivity, while mast cell sensitivity peaked later at 8 weeks.

The 4‐week serum sIgE peak is consistent with previous studies [10, 11, 23, 34, 35] and was accompanied by a parallel increase in sIgG, although sIgG, sIgG1 and sIgG4 levels peaked 2 weeks later than sIgE. Serum and nasal sIgE kinetics were closely aligned. Though local IgE production in the nose [36, 37] and changes in nasal sIgE during season exposure [38, 39] are well established, the duration of its persistence and its contribution to systemic sIgE levels remain unclear. The strong association between nasal and serum sIgE, sIgG1 and sIgG4 may suggest that nasal in situ sIgE, sIgG1 and IgG4 production plays an important role in the first weeks following allergen exposure and likely occurs alongside immune responses in local lymphoid tissues. The rise in sIgG1 and sIgE could equally support the hypothesis that secondary sIgE production occurs through sequential class switching from sIgG1 [40, 41]. This hypothesis is supported by recent data from patients with local allergic rhinitis undergoing NC with allergen, where sequential ε‐switch circles in nasal biopsies have been observed following allergen exposure [42]. However, in our cohort of volunteers with birch induced AR, the later peak of sIgG1 would speak against this hypothesis. Two recent studies have also challenged this and showed high‐affinity IgE can be produced independently of IgG1 intermediates [43, 44] which supports the interpretation that clonal IgE responses following nasal allergen exposure are not exclusively dependent on IgG1‐to‐IgE class switching. At the same time, we acknowledge that serum isotype kinetics alone are an imprecise proxy for B‐cell lineage relationships, as sIgE and sIgG1 may be generated in parallel by distinct B‐cell populations and anatomical compartments with differing output kinetics. In this context, it is conceivable that the early IgE peak reflects reactivation of pre‐existing IgE^+^ plasmablasts [45, 46, 47] which have been reported to be elevated in allergic individuals, whereas allergen‐specific IgG production may depend on reactivation of memory B cells or the initiation of new vaccination‐like immune responses and therefore appears later in the serum. Importantly, this interpretation remains speculative because our analysis was limited to circulating immunoglobulin levels and did not include deep sequencing of blood‐ or nasal‐derived B cells, precluding assessment of the underlying cellular sources. Taken together, these observations suggest possible differences in IgE class‐switching dynamics across allergic rhinitis phenotypes, warranting further study.

Interestingly, we noticed that sIgA increased only in nMLF but not in serum, indicating local sIgA production induced by the high dose allergen exposure. In a prior analysis of the same cohort [16], we noted a trend toward elevated nasal B‐cell activating factor (BAFF) and A proliferation‐inducing ligand (APRIL) levels in allergen provoked patients. Given that both cytokines are known to drive IgA class switching [48], this finding provides mechanistic context that could support this hypothesis, but the relatively rapid appearance of allergen‐specific IgA after provocation is also compatible with activation of pre‐existing local IgA memory B cells. Furthermore, no such induction was found after seasonal allergen exposure. Thus, the induction of sIg responses by NC with allergen appeared to stimulate some immunological features to those seen in the course of sublingual allergen immunotherapy (SLIT), such as increases in sIgE, sIgG and sIgA responses [49, 50, 51]. Whether this may ultimately lead to clinical tolerance if dosed appropriately remains to be determined. The role of IgG and IgA in inhibiting allergic responses is an intriguing subject [15]. Both IgG1 and IgG4 are known to bind to the inhibitory receptor FcγRIIb and downregulate basophil activation [52, 53]. Furthermore, IgA and IgG, particularly IgG4, can act as a blocking antibody by competing with IgE for allergen binding and is regarded as a hallmark of a successful allergen immunotherapy (AIT), correlating with clinical responsiveness [54, 55] and other work demonstrated that the induction of blocking antibody in patients receiving subcutaneous AIT correlated with the allergen concentration tolerated following NC confirms their neutralising ability [56]. This reduced availability of binding sites for IgE by steric hindrance through IgG also results in reduced binding of allergen‐IgE complexes to CD23, which activates T cells via facilitated allergen presentation (FAP) [57, 58]. The increase of sIgG1 and sIgG4 as well as locally produced IgA we observed might conceivably contribute to the dampened IgE response during seasonal allergen exposure due to reduced T‐cell activation. In this light, it has been reported in several immunotherapy studies that treated patients showed reduced boosts of seasonally induced IgE responses [59, 60, 61]. Therefore, these findings may provide some valuable insights into potential mechanisms in AIT.

During the seasonal part of the study, only the placebo group displayed increases in sIgE, suggesting a blunted immune response in the BPE group. However, this attenuation did not translate into measurable differences in clinical symptom burden between the two groups during the pollen season, most likely due to group size, differences in CSMS “baseline” score prior to detectable pollen counts, or that the observed immunological differences were too subtle to translate into measurable clinical differences. Nevertheless, the observed immunological pattern may provide important insights into potential mechanisms of immune modulation and tolerance that could inform future investigations. In this context, it is still a matter of debate whether previous allergen exposure could ‘prime’ the immune system to trigger a greater immune response with subsequent provocation. Our data support an absence of priming [62], though this may depend heavily on timing and amount of allergen [63]. In fact, we also found that short‐term repeated allergen exposure during the NC with allergen resulted in reduced nasal sensitivity on the third day of challenge as compared to the first day. This is in contrast to studies reporting that repeated NC with allergen results in enhanced nasal sensitivity [64]. However, there are also studies suggesting that nasal sensitivity may remain unaffected or decrease after repeated NC with allergen [65, 66]. In this context, it should not be forgotten that it is possible to desensitise basophils and allergic effector cells in an antigen‐specific manner, and rush immunotherapy has been reported to be effective before induction of blocking antibodies, presumably due to ‘tachyphylaxis’ [67, 68, 69, 70]. The fact that NC with allergen but not natural seasonal allergen exposure induced increases of sIgE and sIgG in the BPE NC group, despite a particularly strong birch pollen season during the study period, is in line with previous reports [39, 71] and can most likely be explained by different exposure kinetics and effective mucosal dose between the two settings. Unlike the nasal challenge, which delivered a controlled high‐dose bolus to the nasal mucosa over consecutive days and likely triggered strong local immune activation, natural pollen exposure is variable and intermittent, with lower allergen deposition, enough to cause symptoms and IgE rises in the serum of patients with no out of season NC but not necessarily to reproducibly boost local Bet v 1 antibodies in nasal secretions. In addition, antigen dose may influence the activation of allergen‐specific memory responses, and a sufficiently high local allergen dose may be required to boost pre‐existing IgE memory cells within the nasal mucosa [72]. This also raises the possibility that repeated nasal allergen exposure, if applied at higher doses and with more repetitions, could potentially influence IgE responses due to blocking antibodies as has been shown in local nasal immunotherapy studies [73]. Finally, the absence of a significant nasal IgE boost during natural exposure on the BPE group may also reflect the induction of regulatory pathways, as repeated allergen contact can promote IL‐10–producing regulatory B and T cells that suppress IgE production [74] and favour IgG4 production, a mechanism well described in allergen immunotherapy settings [75].

Following NC with allergen, basophil sensitivity increased concurrently with the peak in sIgE, whereas mast cell sensitivity peaked 2–4 weeks later. Whilst previous in vivo studies were mainly investigating desensitisation of basophil and mast cells by IgE depletion or complexing observed at 4–12 weeks [13, 14, 76], so far the direct impact of IgE on modulating FcεRI levels and hence mediator release has only been demonstrated in vitro [77, 78, 79]. The kinetics of sensitization observed in our study are slightly faster than the 4–12 weeks reported for desensitisation [13, 14, 76], this may also be partially attributed to our significantly shorter sampling intervals compared to previous studies, particularly in relation to mast cell sensitivity. Furthermore our longitudinal data of course reflect the process in systemic AR but recent work using NAC to define rhinitis phenotypes into AR, local allergic rhinitis, dual allergic rhinitis and non‐allergic rhinitis has shown that BAT responses themselves differ across these endotypes [80]. Differences in basophil and mast cell sensitivity may be due to differences in receptor turnover, localisation and lifespan: while tissue resident mast cells may survive several months, circulating basophils live only for a few days [81]. It should be noted that different allergen formulations were used in BAT (recombinant Bet v 1) and SPT (birch pollen extract). However, recombinant Bet v 1 has been shown to be immunologically equivalent to natural Bet v 1 [82], and within each assay system the same allergen preparation was used across all time points. Therefore, differences in allergen formulation are unlikely to explain the delayed kinetics observed. The reason we only observed transient changes in basophil sensitivity in our study is likely multifactorial. Basophil activation depends on IgE affinity and epitope diversity, which influence cross‐linking efficiency and activation thresholds [83, 84]. Furthermore FcεRI expression on basophils is also dynamically regulated by IgE availability and receptor turnover, such that changes in basophil sensitivity may be decoupled from changes in circulating IgE [76, 77, 85]. In addition, induction of sIgG, particularly sIgG4, can reduce basophil activation by competing with IgE for allergen and by engaging inhibitory FcγRIIb pathways, which may attenuate BAT changes even when IgE rises [86, 87].

Previous work from our group and others have shown that strong rises of IL‐13 2–8 h after allergen exposure correspond with subjects that develop a clinical late allergic reaction in the context of AR [16, 17, 18]. These subjects have higher detectable levels of type 2 inflammatory cytokines in their noses and we therefore hypothesised that this could affect their systemic allergic immune responses in the weeks after allergen exposure. Indeed, we saw this to hold true for sIgE in the serum, in which greater rises from baseline were seen in the IL‐13^R^ group and gained significance in IL‐13^R^ vs. IL‐13^NR^ at 8–12 weeks after nasal allergen provocation. We also observed non‐significant trends for greater mast cell and basophil sensitivity in the IL‐13^R^ group. As far as we know, this has not been investigated before in the context of allergic rhinitis but in allergic asthma high circulating IgE levels are correlated with late asthmatic responses [88, 89, 90, 91]. Furthermore IL‐13 produced by T follicular helper (Tfh) cells in mice and humans has been shown to increase IgE production and affinity [92]. These findings, taken together with our data, may suggest that cytokines promoting late phase responses, such as IL‐5 and IL‐13, influence the strength of the systemic immune response even weeks and months after allergen exposure. Nevertheless, it is important to note that these findings are purely exploratory and this study is too underpowered to draw any mechanistic conclusions and will need to be confirmed in larger prospective studies.

The relatively small cohort size can be considered a limitation of this study; however, compared to previous provocation studies, our sample size was similar. Furthermore, the sample size was adequately powered to ensure that changes in immunoglobulin responses could be detected. Additionally, subjective NC induced ocular symptoms were not separately assessed during the post challenge time course, which are common post NC, but the burden of these symptoms will have contributed to the total symptom burden VAS. A further limitation relates to the quantification of allergen‐specific antibodies by ELISA. Absolute concentration standards were available only for Bet v 1‐specific IgE and IgG4, whereas IgA, IgG, and IgG1 responses were assessed using optical density measurements, limiting direct comparison of absolute concentrations with other studies. However, as the primary aim of the antibody analyses was to characterise within‐subject temporal dynamics, all time points from each participant were analysed on the same ELISA plate and responses were expressed as log_2_ fold changes relative to baseline, enabling robust comparison of longitudinal antibody kinetics within the study cohort. Furthermore, a limitation of the seasonal follow‐up is reliance on daily self‐reported CSMS diaries and a limited number of visits during and after the season, which may reduce temporal resolution and introduce reporting bias, although regular reminders were used to mitigate recall bias and visits were timed to capture key seasonal responses. Strengths of our pilot study, however, lie in its intricate design, including regular assessment of effector cell sensitivity, novel data, and a high volunteer retention rate.

In summary, our study provides key and novel insights into the interaction of systemic sIg responses in the serum and nose with changes in cellular sensitivity following NC with allergen. These observed kinetics will provide valuable insights for future therapeutic studies, aiding in the identification of critical time windows to evaluate therapeutic effects and enhance intervention strategies for emerging treatments.

## Author Contributions

R.V., V.N.‐L., J.E.‐D., N.J.C. and R.R. designed the study. J.E.‐D., V.N.‐L., R.V. and N.J.C. wrote the study protocol and gained ethical approval for the study. J.E.‐D. and N.J.C. recruited all the volunteers and J.E.‐D., N.J.C., S.V.‐M., V.S., J.T., K.G. and S.S. carried out the study. N.J.C., J.E.‐D., V.S., S.V.‐M., A.T., M.F.‐T., R.F., G.R. and T.P. performed the experiments. N.J.C., J.E.‐D., S.V.‐M., H.B., M.B., R.V., V.N.‐L. and C.M. performed the analysis. N.J.C., J.E.‐D., M.Z., C.M., R.V. and V.N.‐L. wrote the manuscript. All authors critically revised the manuscript and approved its final version together.

## Funding

This work was supported by grants DK W 1248‐B30, SFB F4613, P34472‐B and KLP4891723 from the Austrian Science Fund (FWF) and by the DANUBE ARC programme of the County of Lower Austria.

## Conflicts of Interest

R.V. serves as a lecturer for HVD Biotech, Vienna, Austria. J.E.‐D. and S.S. have received research grants from AstraZeneca, Cambridge, UK and AstraZeneca, Austria as well as Sanofi and GSK.

## Supporting information


**Table S1:** Patient demographics and sensitization profile.

**Table S2:** Patient baseline antibody and effector responses.

**Table S3:** BAT sensitivity and reactivity.

**Figure S1:** Study consort statement of volunteers screened and participating in the study. Figure created in https://BioRender.com.

**Figure S2:** Changes in Bet v 1 specific IgE as determined by ImmunoCAP in serum of birch pollen allergic subjects provoked with placebo (*n* = 10) or birch pollen extract (BPE, *n* = 20).

**Figure S3:** Dynamics of the Bet v 1–specific IgE and basophil effector response.

**Figure S4:** Changes in IgG1, IgG4, IgA, before and after birch seasonal exposure and BAT at 8 weeks and 20 weeks post season.

**Figure S5:** Selected allergen‐specific serum Ig responses, changes in basophil activation test (BAT) and titrated skin prick test (tSPT) in volunteers with (IL‐13R, red, *n* = 8) or without late phase allergic IL‐13 response (IL‐13NR, yellow, *n* = 12) during birch pollen extract (BPE) provocation vs. those being provoked with placebo (Placebo, grey, *n* = 10).

**Figure S6:** Changes in PNIF and IL‐13 during visit N1 in volunteers with (IL‐13R, red, *n* = 8) or without late phase allergic IL‐13 responses (IL‐13NR, yellow, *n* = 12) after birch pollen extract provocation vs. those being provoked with placebo (Placebo, grey, *n* = 10).

**Figure S7:** Changes in Bet v 1 specific IgE, IgG1 and IgG4 in nMLF in volunteers with (IL‐13R, red, *n* = 8) or without late phase allergic IL‐13 responses (IL‐13NR, yellow, *n* = 12) after birch pollen extract provocation vs. those being provoked with placebo (Placebo, grey, *n* = 10).

**Figure S8:** Selected clinical and immunological responses to seasonal allergen exposure in relation to IL‐13 responder status in the seasonal arm of the study. IL‐13 responder (IL‐13R, red, *n* = 8) vs. IL‐non‐responder 13 response (IL‐13NR, orange, *n* = 12) vs. placebo (Placebo, grey, *n* = 10).

**Data S1:** Supplementary methodology.

## Data Availability

The data that support the findings of this study are available on request from the corresponding author. The data are not publicly available due to privacy or ethical restrictions.
