# Supplementary material for: Kinetics of Antibody Responses and Effector Cell Sensitivity After High Dose Birch Extract Nasal Challenge
Source: Allergy. 2026 May 4;81(11):3920–35. doi: 10.1111/all.70365 (PMC13753670; doi:10.1111/all.70365)
Supplement: Supplementary file 1 [file ALL-81-3920-s001.pdf]

Figure S2

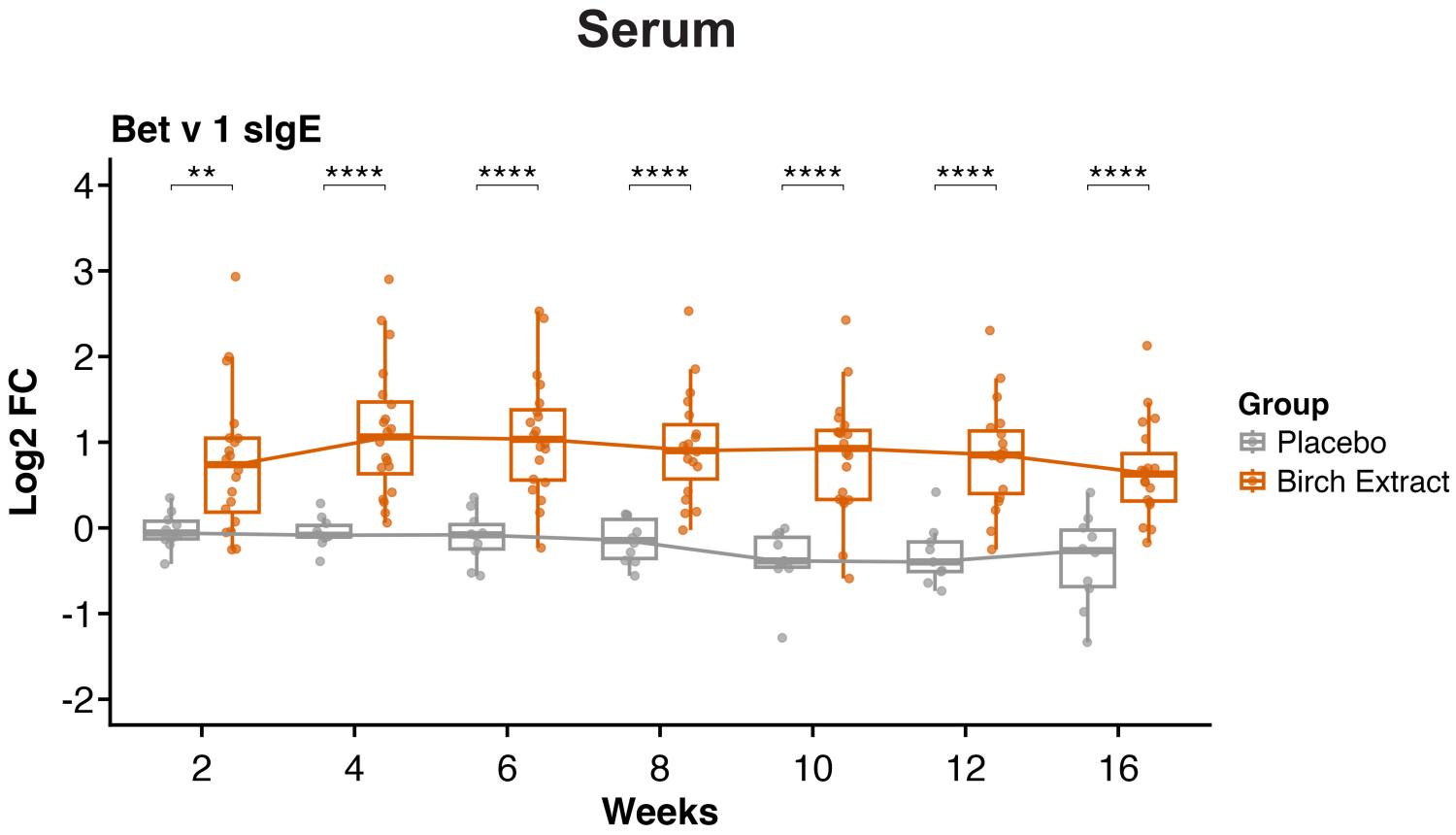

Serum
Bet v 1 sIgE
**
****
****
****
****
****
****
4
3
2
1
0
-1
-2
Log2 FC
2
4
6
8
10
12
16
Weeks
Group
Placebo
Birch Extract
