# Supplementary material for: Kinetics of Antibody Responses and Effector Cell Sensitivity After High Dose Birch Extract Nasal Challenge
Source: Allergy. 2026 May 4;81(11):3920–35. doi: 10.1111/all.70365 (PMC13753670; doi:10.1111/all.70365)
Supplement: Supplementary file 2 [file ALL-81-3920-s002.pdf]

Figure S5

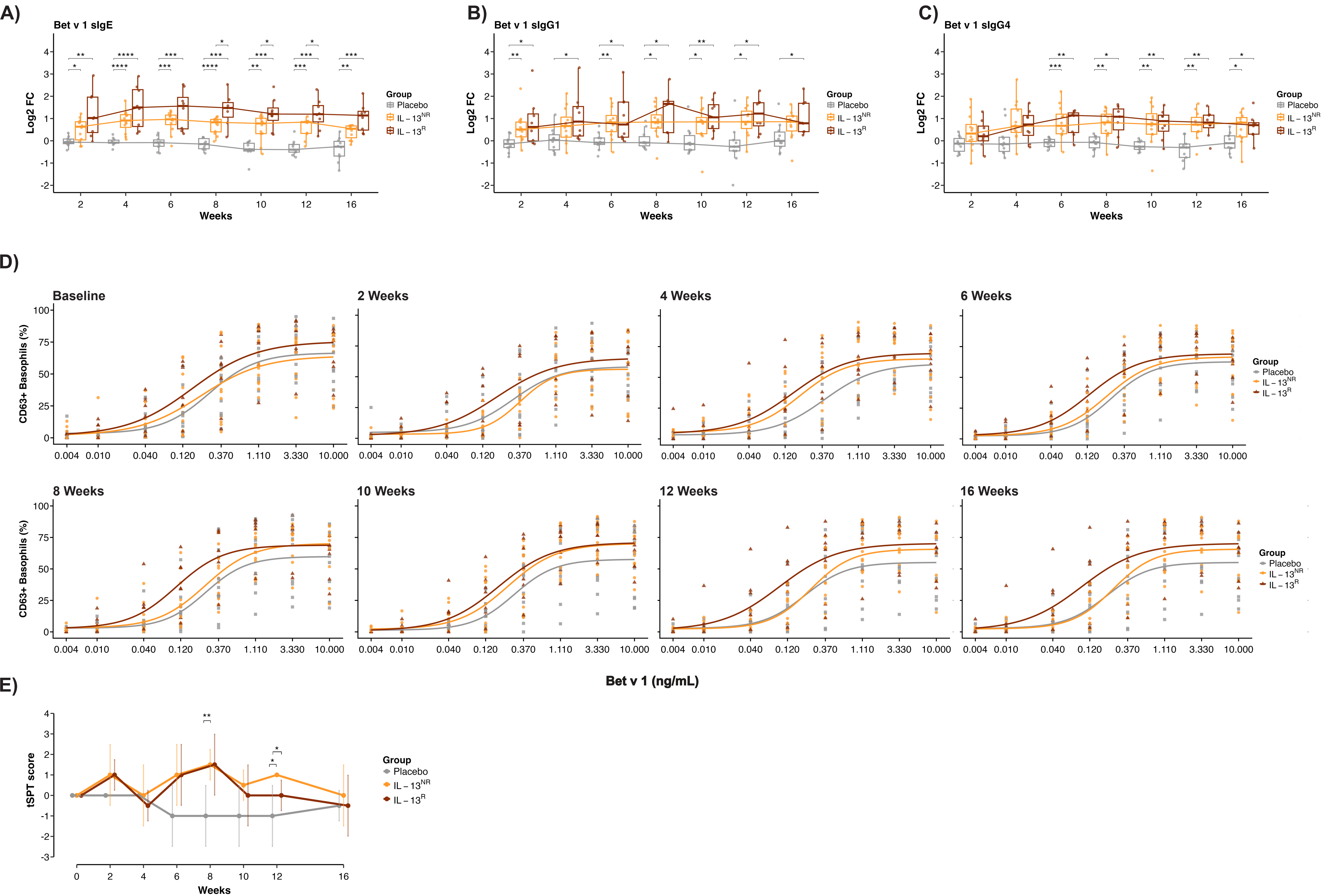

A)
Bet v 1 sIgE
Log2 FC
Weeks
B)
Bet v 1 sIgG1
C)
Bet v 1 sIgG4
Group
Placebo
IL – 13NR
IL – 13R
D)
Baseline
2 Weeks
4 Weeks
6 Weeks
8 Weeks
10 Weeks
12 Weeks
16 Weeks
CD63+ Basophils (%)
0.004 0.010 0.040 0.120 0.370 1.110 3.330 10.000
Bet v 1 (ng/mL)
E)
tSPT score
Weeks
