# Supplementary material for: Kinetics of Antibody Responses and Effector Cell Sensitivity After High Dose Birch Extract Nasal Challenge
Source: Allergy. 2026 May 4;81(11):3920–35. doi: 10.1111/all.70365 (PMC13753670; doi:10.1111/all.70365)
Supplement: Supplementary file 3 [file ALL-81-3920-s003.pdf]

Figure S6

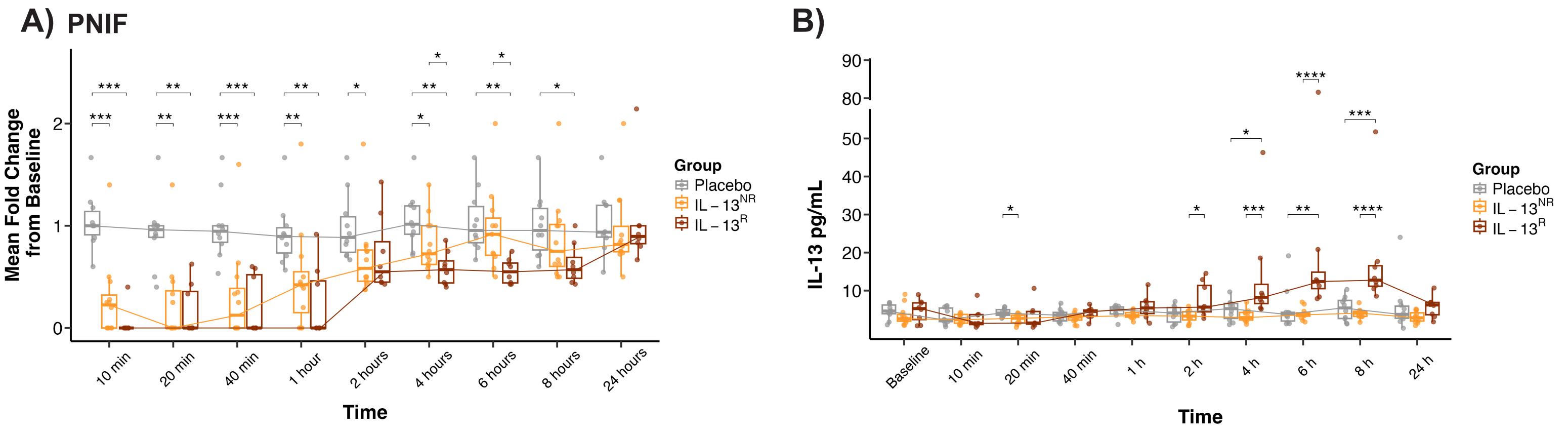

A) PNIF
Mean Fold Change from Baseline
0
1
2
***
**
*
10 min
20 min
40 min
1 hour
2 hours
4 hours
6 hours
8 hours
24 hours
Time
Group
Placebo
IL – 13NR
IL – 13R
B)
IL-13 pg/mL
10
20
30
40
50
80
90
Baseline
10 min
20 min
40 min
1 h
2 h
4 h
6 h
8 h
24 h
Time
****
Group
Placebo
IL – 13NR
IL – 13R
