# Supplementary material for: Kinetics of Antibody Responses and Effector Cell Sensitivity After High Dose Birch Extract Nasal Challenge
Source: Allergy. 2026 May 4;81(11):3920–35. doi: 10.1111/all.70365 (PMC13753670; doi:10.1111/all.70365)
Supplement: Supplementary file 4 [file ALL-81-3920-s005.pdf]

Figure S8

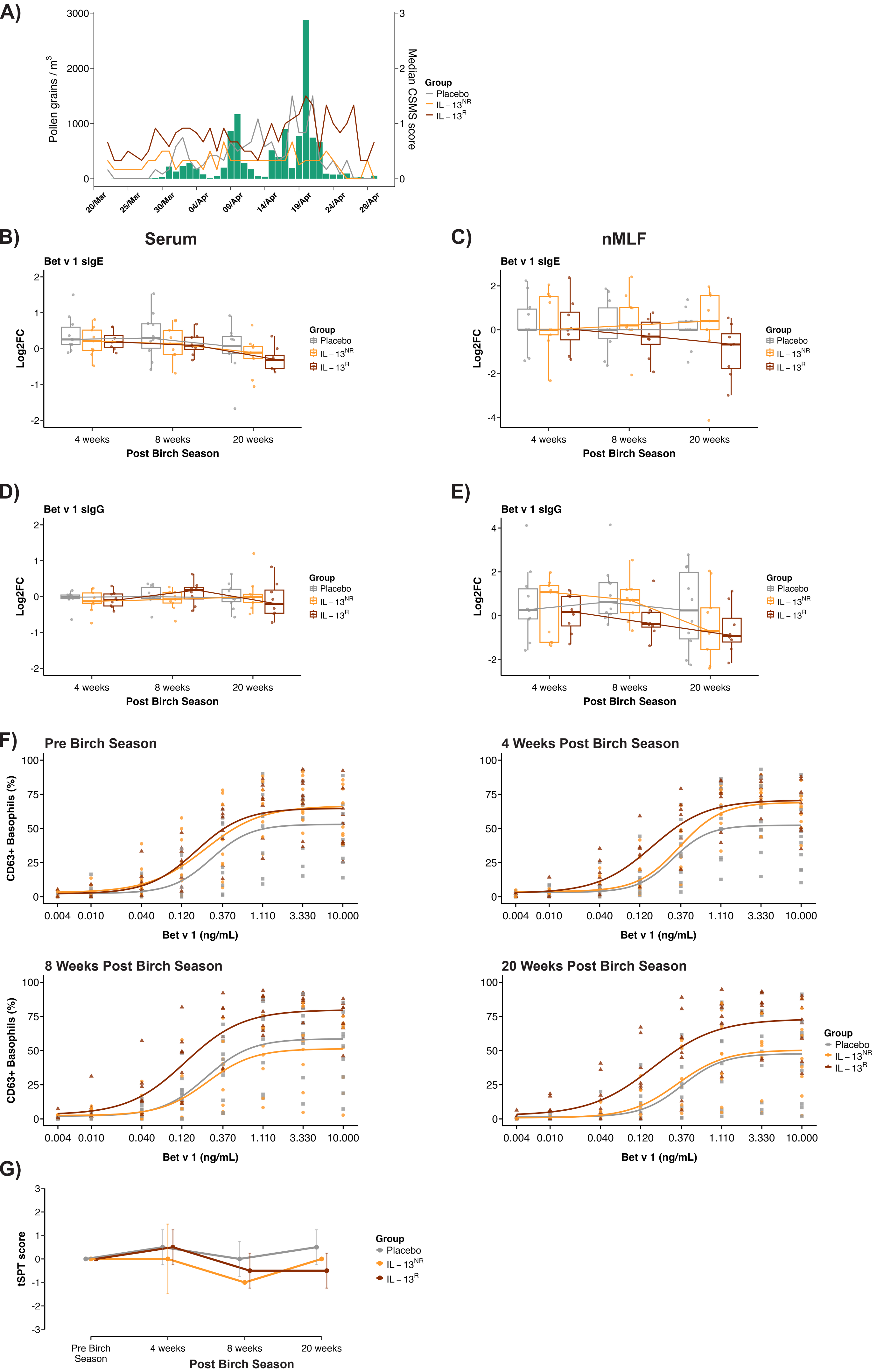
A)
Pollen grains / m3
Median CSMS score
Group
Placebo
IL – 13NR
IL – 13R
20/Mar
25/Mar
30/Mar
04/Apr
09/Apr
14/Apr
19/Apr
24/Apr
29/Apr
B)
Serum
C)
nMLF
Bet v 1 sIgE
Log2FC
4 weeks
8 weeks
20 weeks
Post Birch Season
D)
E)
Bet v 1 sIgG
F)
Pre Birch Season
4 Weeks Post Birch Season
8 Weeks Post Birch Season
20 Weeks Post Birch Season
CD63+ Basophils (%)
Bet v 1 (ng/mL)
G)
tSPT score
Pre Birch Season
