# Supplementary material for: Kinetics of Antibody Responses and Effector Cell Sensitivity After High Dose Birch Extract Nasal Challenge
Source: Allergy. 2026 May 4;81(11):3920–35. doi: 10.1111/all.70365 (PMC13753670; doi:10.1111/all.70365)
Supplement: Supplementary file 5 [file ALL-81-3920-s008.pdf]

Figure S3

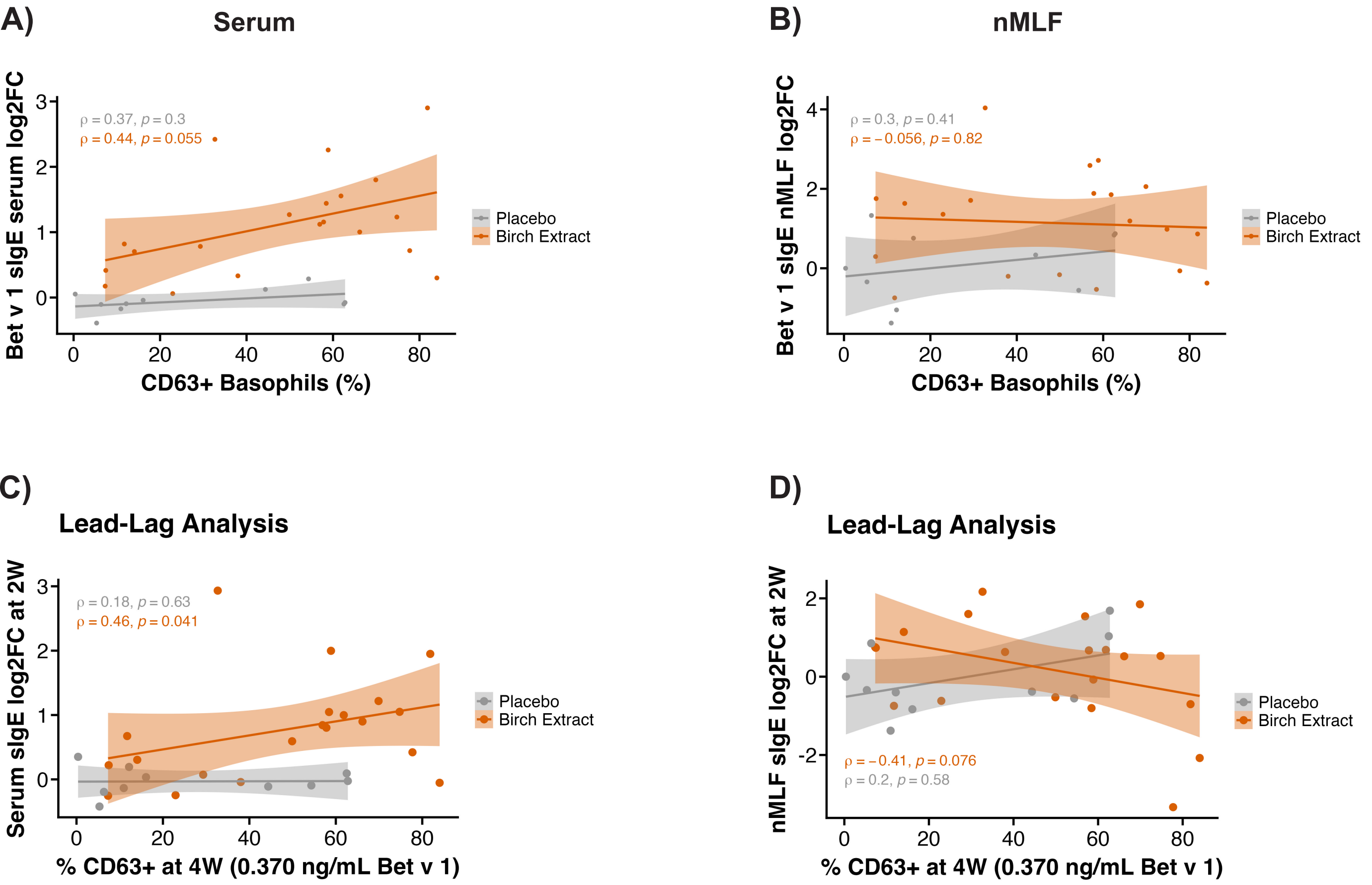

A)
Serum
ρ = 0.37, p = 0.3
ρ = 0.44, p = 0.055
Bet v 1 sIgE serum log2FC
CD63+ Basophils (%)
Placebo
Birch Extract
B)
nMLF
ρ = 0.3, p = 0.41
ρ = −0.056, p = 0.82
Bet v 1 sIgE nMLF log2FC
CD63+ Basophils (%)
Placebo
Birch Extract
C)
Lead-Lag Analysis
ρ = 0.18, p = 0.63
ρ = 0.46, p = 0.041
Serum sIgE log2FC at 2W
% CD63+ at 4W (0.370 ng/mL Bet v 1)
Placebo
Birch Extract
D)
Lead-Lag Analysis
ρ = −0.41, p = 0.076
ρ = 0.2, p = 0.58
nMLF sIgE log2FC at 2W
% CD63+ at 4W (0.370 ng/mL Bet v 1)
Placebo
Birch Extract
