# Supplementary material for: Kinetics of Antibody Responses and Effector Cell Sensitivity After High Dose Birch Extract Nasal Challenge
Source: Allergy. 2026 May 4;81(11):3920–35. doi: 10.1111/all.70365 (PMC13753670; doi:10.1111/all.70365)
Supplement: Supplementary file 6 [file ALL-81-3920-s009.pdf]

Figure S1

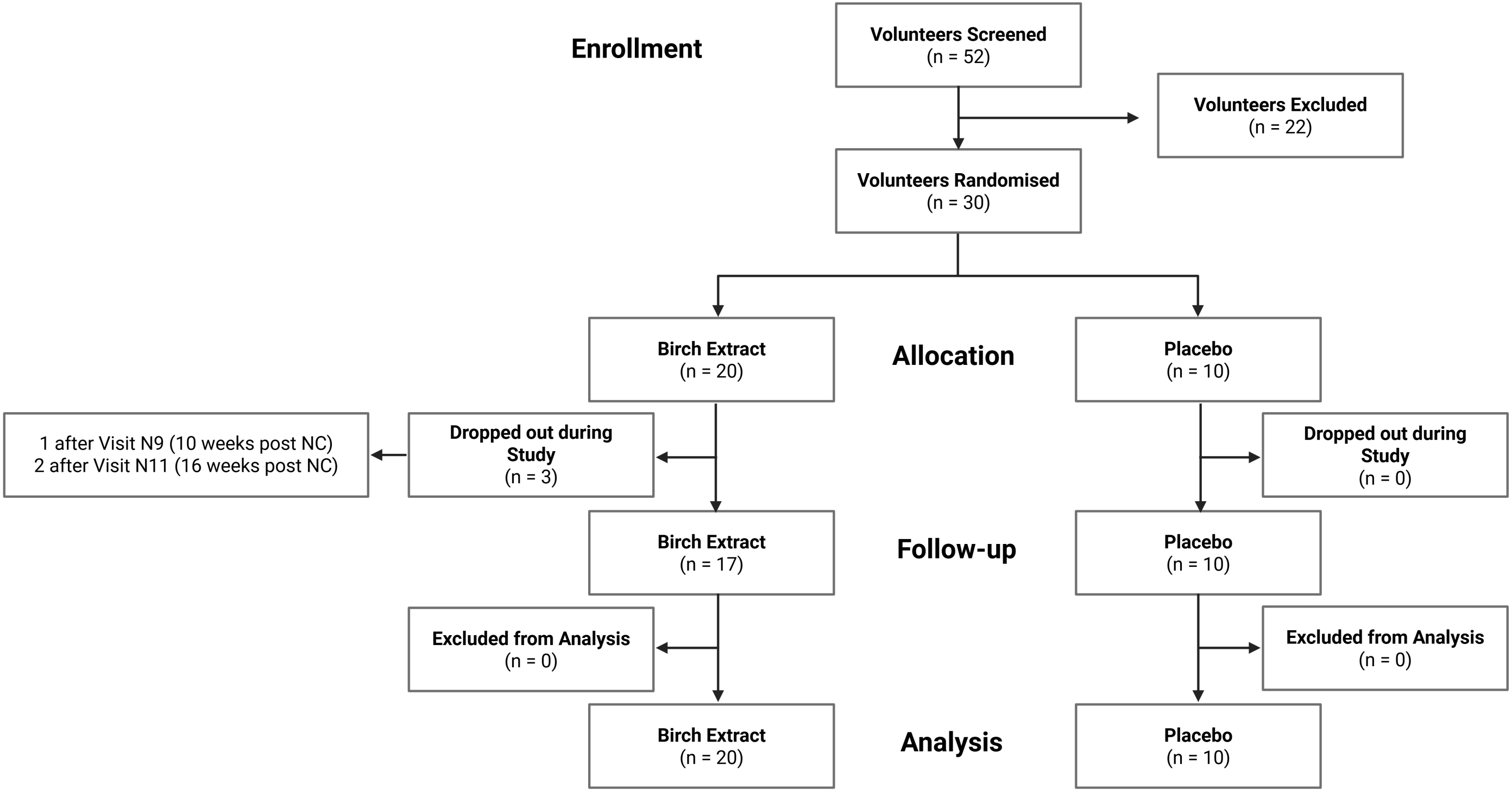

Enrollment
Volunteers Screened
(n = 52)
Volunteers Excluded
(n = 22)
Volunteers Randomised
(n = 30)
Birch Extract
(n = 20)
Allocation
Placebo
(n = 10)
1 after Visit N9 (10 weeks post NC)
2 after Visit N11 (16 weeks post NC)
Dropped out during Study
(n = 3)
Dropped out during Study
(n = 0)
Birch Extract
(n = 17)
Follow-up
Placebo
(n = 10)
Excluded from Analysis
(n = 0)
Excluded from Analysis
(n = 0)
Birch Extract
(n = 20)
Analysis
Placebo
(n = 10)
