# Supplementary material for: Kinetics of Antibody Responses and Effector Cell Sensitivity After High Dose Birch Extract Nasal Challenge
Source: Allergy. 2026 May 4;81(11):3920–35. doi: 10.1111/all.70365 (PMC13753670; doi:10.1111/all.70365)
Supplement: Supplementary file 7 [file ALL-81-3920-s011.pdf]

Figure S4

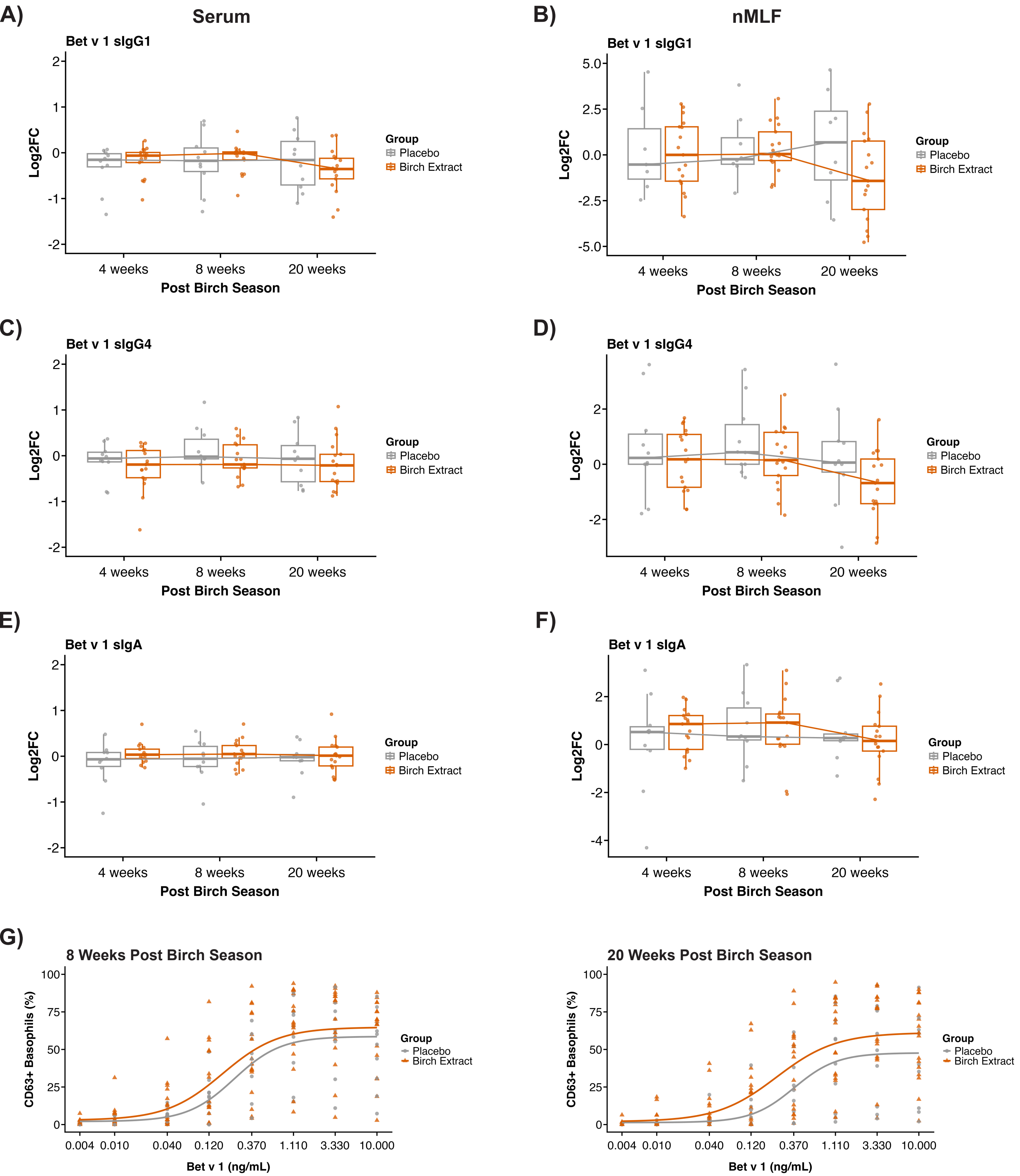

A)
Serum
Bet v 1 sIgG1
Log2FC
2
1
0
-1
-2
4 weeks
8 weeks
20 weeks
Post Birch Season
Group
Placebo
Birch Extract
B)
nMLF
Bet v 1 sIgG1
5.0
2.5
0.0
-2.5
-5.0
C)
Bet v 1 sIgG4
D)
Bet v 1 sIgG4
E)
Bet v 1 sIgA
F)
Bet v 1 sIgA
-4
G)
8 Weeks Post Birch Season
20 Weeks Post Birch Season
CD63+ Basophils (%)
100
75
50
25
0
0.004
0.010
0.040
0.120
0.370
1.110
3.330
10.000
Bet v 1 (ng/mL)
