# Supplementary material for: Kinetics of Antibody Responses and Effector Cell Sensitivity After High Dose Birch Extract Nasal Challenge
Source: Allergy. 2026 May 4;81(11):3920–35. doi: 10.1111/all.70365 (PMC13753670; doi:10.1111/all.70365)
Supplement: Supplementary file 9 [file ALL-81-3920-s004.docx]

**ONLINE REPOSITORY**

**Kinetics of Antibody Responses and Effector Cell Sensitivity after High Dose Birch Extract Nasal Challenge**

N.J. Campion MBChB, PhD^a^, S. Villazala-Merino PhD^a^, C. Morgenstern PhD^a^, Nemec L^a^, V. Stanek MSc^a^, Aldine Tu MSc^a^, M. Zghaebi PhD^a^, Bastl M^a^, J. Toth MD^a^, R. Fröschl^b^, T. Perkmann MD^b^, M. Bastl^a^, PhD, K. Gangl MD^a^, S. Schneider MD^a^, H. Breiteneder PhD^d^, R. Ristl PhD^c^, M. Focke-Tejkl PhD^d^, R. Valenta MD^d,e^ , V. Niederberger-Leppin MD^a*^ and J. Eckl-Dorna MD, PhD^a^

**AUTHOR AFFILIATIONS**

^a^Vienna Airway Lab, Department of Otorhinolaryngology, Head and Neck Surgery, Medical University of Vienna, Vienna, Austria

^b^Department of Laboratory Medicine, Medical University of Vienna, Vienna, Austria

^c^Center for Medical Statistics, Informatics and Intelligent Systems, Medical University of Vienna, Vienna, Austria

^d^Divison of Immunopathology, Department of Pathophysiology and Allergy Research, Center for Pathophysiology, Infectiology and Immunology, Medical University of Vienna, Vienna, Austria

^e^Center for Molecular Allergology, Karl Landsteiner University of Health Sciences, Krems, Austria

**CORRESPONDING AUTHOR**

Julia Eckl-Dorna, MD, PhD

Department of Otorhinolaryngology

Research laboratories 8H

Medical University of Vienna, General Hospital of Vienna

Waehringer Guertel 18-20

A-1090 Vienna, Austria

Tel: +43-1-40400-34380

Email: [julia.eckl-dorna@meduniwien.ac.at](mailto:julia.eckl-dorna@meduniwien.ac.at)

**Supplementary Methods**

**1.1 Study Design:** This study was performed at a single centre at the University Clinic of Otorhinolaryngology of the Medical University of Vienna. To avoid the inclusion of participants with nasal hypersensitivity, patients were excluded, if they showed significant discomfort or immediately sneezed after the application of nasosorptions (Nasosorption FX-I, Hunt Developments (UK) Limited, Midhurst, West Sussex, United Kingdom) to avoid potential confounders by including patients with non-allergic rhinitis.

**1.2 Pre-specified and exploratory outcomes**

The primary objective of the study was to assess the relationship between allergen-specific IgE production and changes in basophil and mast cell sensitivity following nasal exposure to birch pollen extract in birch-allergic subjects. Pre-specified secondary outcomes included longitudinal changes in allergen-specific IgE levels in serum and nasal secretions, changes in allergen-specific IgA, IgG, IgG1, and IgG4 levels in serum and nasal secretions, assessment of subjective disease burden using the total nasal symptom score (TNSS) and combined symptom medication score (CSMS) and objective assessment of nasal obstruction using peak nasal inspiratory flow (PNIF). Planned analyses of cytokine and mediator patterns during the nasal challenge component of the study were also planned at study design and were performed and have been reported separately (1). In addition to the pre-specified analyses, exploratory post hoc analyses stratifying participants by IL-13 responder status were conducted based on observations from previous work in this cohort (1) and are presented as hypothesis-generating analyses.

***1.3* Screening Skin Prick and Titrated (t) Test:** The prick test for screening was performed on the volunteer’s forearms as previously described (2, 3). Specifically, birch, alder, ash, grass, rye, mugwort, ragweed, *D. pteronyssinus*, *D. farinae*, dog, cat, guinea pig, *Alternaria*, *Cladosporium* and A*spergillus* were tested for. For the tSPT the clinicians and patients were blinded to the content of the skin prick test solutions and the randomization scheme. The solutions were prepared the same day they were to be used by our lab technician not involved in performing the tSPT. They were prepared and stored in glass vials. After 20 minutes circles were drawn around wheals of any positive reaction and transferred to the study data files with cello tape. The wheels were then scanned and analysed by ImageJ (U. S. National Institutes of Health, Bethesda, Maryland, USA) to determine wheel area in mm^2^. Using quadruplicates at different positions of the forearm, we observed inter individual differences. Thus, to select only true positive reactions for analysis, the following strict criteria were applied:

A positive reaction to a particular concentration was considered if:

1) at least ¾ of the replicates had measurable wheals
2) the average wheal area across all 4 quadruplicates was >7mm^2^ which corresponds to the internationally accepted cutoff of a wheel diameter of 3mm.
3) at least 1 of the 2 preceding concentrations had positive reactions based on these first two criteria.
4) Lastly, to account for a “tailing off” effect, if, at the final concentration of reactivity, a volunteer showed a measurable reaction in only 2 out of 4 replicates, it was still considered a positive reaction as long as the previous concentration met the criteria mentioned earlier, and the average wheal area was greater than 7mm². This exception was only applied during the final concentration a volunteer reacted to.

**1.4 Serum and nMLF Ig Measurements by ELISA:** Nunc® MaxiSorp™ 384 well plates (Merck, Rahway, New Jersey, USA) were coated with recombinant (r)Bet v 1 at 1µg/ml (Biomay, Vienna, Austria) in 100mM carbonate/bicarbonate buffer (Sigma-Aldrich, St. Louis, Missouri, USA) at 4^o^C overnight. Plates were then washed 5 times with PBST (PBS (Morphisto, Offenbach, Germany) 0.05% Tween 20 (Sigma Life Sciences, Whitby, Ontario, Canada)) and then blocked with PBST 2% BSA (Sigma-Aldrich, St Louis, Missouri, USA) for 2 hours at room temperature. Serum and nMLF samples were diluted in 0.5% BSA in PBST as follows for detection of the different isotypes: IgE 1/2, IgA 1/5 and IgG, G1 and G4 1/10 and were added to the plates after washing. For IgE the CB1 IgE standard produced as previously described (4) was used. 8 ½ dilution steps of CB1 IgE starting at 100ng/ml were used to generate a linear standard curve from 0.8-100ng/ml. Bet v 1–specific IgG4 came from the human monoclonal Bet v 1–specific IgG4 antibody BAB5 (5) following the approach for quantitative allergen-specific IgG4 ELISA using monoclonal antibody standards as described previously (6). . 8 one in two dilution steps of BAB5 IgG4 starting at 1000ng/ml were used to generate a linear standard curve from 7.8-1000ng/ml. Plates were incubated overnight at 4°C. After washing, the following secondary antibodies were added: anti-IgE (BD Pharmigen, New Jersey, USA), anti-IgA (BD Pharmigen, New Jersey, USA), anti-IgG (BD Pharmigen, New Jersey, USA), anti-IgG1 (BD Pharmigen, New Jersey, USA) and anti-IgG4 (BD Pharmigen, New Jersey, USA) all at 1:1000 dilution and incubated at 4^o^C overnight. For IgG1, 4 and IgA streptavidin HRP (BD Pharmigen, New Jersey, USA) needed to be added at 1:1000 dilution for one hour after washing. After this plates were washed and 20µl of ABTS (Sigma Life Sciences, Whitby, Ontario, Canada) (used for IgA, IgG, IgG1 and IgG4) or AKP (Sigma Life Sciences, Whitby, Ontario, Canada) (for IgE) were added and samples read at 410nm. ELISA measurements were performed in triplicates. To minimize plate-to-plate variability, all timepoints for a given patient were consistently run on the same plate across all visits and the same sample mixed of sera and nMLF from different patients was measured on each plate to ensure comparability.

**1.5 Environmental Pollen Concentrations and Combined Symptom Medication Scores:** Pollen concentrations were measured during the season via a volumetric pollen and spore trap of the Hirst 1 design located on the rooftop of the National Austrian Meteorological Service, Vienna (Zentralanstalt für Meteorologie und Geodynamik, ZAMG, 48°14’57’’N 16°21’24’’E) at an elevation of 14m.
During the birch pollen season, volunteers were asked to keep a paper diary of their daily combined symptom medication scores, which was used and calculated as previously described (7). Briefly, four nasal symptoms (rhinorrhea, nasal obstruction, nasal itching, and sneezing) and two ocular symptoms (itchy eyes and watery/red eyes) were each graded daily on a 4-point scale from 0 to 3 (0 = no symptoms; 1 = mild symptoms; 2 = moderate symptoms; 3 = severe symptoms that are difficult to tolerate). The six symptom scores were summed and divided by six to obtain the daily symptom score (range 0–3). Rescue medication use was graded separately on a 4-point scale: 0 = no medication; 1 = oral or intranasal antihistamines; 2 = intranasal corticosteroids; 3 = oral corticosteroids. The CSMS was calculated as the sum of the daily symptom score (0–3) and the medication score (0–3), resulting in a total score ranging from 0 to 6, with higher scores indicating a greater combined symptom and medication burden.

**1.6 Data and Statistical Analysis:** Data distribution was assessed using the Shapiro-Wilk test and histogram plotting. Non-parametric data were presented as medians with median absolute deviation (MAD) and analysed using Wilcoxon rank-sum tests for independent group-wise comparisons. Benjamini-Hochberg correction was used to adjust for multiple testing. The Kruskal-Wallis rank sum test was applied to compare multiple groups of non-parametric data. The association between two binary variables was assessed using Fisher’s exact test. Statistical significance was assumed at *P* values smaller or equal than 0.05. Figure panels were assembled in Affinity Publisher 2 (2.5.6).

**2. Supplementary Figure Legends:**

**Supplementary Figure 1: Study Consort Statement** of volunteers screened and participating in the study. Figure Created in <https://BioRender.com>

**Supplementary Figure 2: Changes in Bet v 1 specific IgE as determined by ImmunoCAP in serum of birch pollen allergic subjects provoked with placebo (n=10) or birch pollen extract (BPE, n=20).** Boxplots display serum log2 fold changes compared to baseline (x-axis) in IgE across time (y-axis, weeks) in indicated groups following nasal challenge. Horizontal line within box denotes the median, with upper and lower box boundaries representing quartiles and whiskers extending to 1.5 times the interquartile range. Lines connect the median values across time. Asterisks represent statistically significant differences between groups (*: p≤0.05, **: p≤0.01, ***: p≤0.001, ****p≤0.0001). Abbreviations: Ig=immunoglobin

**Supplementary Figure 3: Dynamics of the Bet v 1–specific IgE and basophil effector response. (A, B)** Correlation of Bet v 1–specific serum IgE (sIgE) **(A)** and sIgE in nasal mucosal lining fluid (nMLF) **(B)** at 4 weeks with the basophil activation test (BAT) response (0.370 ng/mL Bet v 1) at 4 weeks. **(C, D)** Correlation of serum sIgE **(C)** and nMLF sIgE **(D)** at 2 weeks with the BAT response (0.370 ng/mL Bet v 1) at 4 weeks. Spearman’s rank correlation coefficient (ρ) and corresponding p-values are shown for each analysis.

Abbreviations: Ig=immunoglobin, nMLF= nasal mucosal lining fluid, 2/4W=2/4 weeks

**Supplementary Figure 4: Changes in IgG1, IgG4, IgA, before and after birch seasonal exposure and BAT at 8 weeks and 20 weeks post season.** Volunteers undergoing nasal provocation with BPE (orange, n= 20) and those being provoked with placebo (grey, n=10). Left panel boxplots **(A, C, E)** show log2 fold changes compared to baseline (y-axis) of Bet v 1 specific IgA **(A)**, IgG1 **(C)**, and IgG4 **(E)** across time (x-axis, weeks) in the serum. Right panel boxplots **(B, D, F)** show log2 fold changes compared to baseline (y-axis) of Bet v 1 specific IgA **(B)**, IgG1 **(D)**, IgG4 **(F)** across time (x-axis, weeks) in nMLF. (G) Changes in BAT sensitivity shown via fitted dose-response curves at 8 weeks (left panel) and 20 weeks post season (right panel) (x-axis, concentration of Bet v 1 exposure in ng/ml, y-axis, % of total basophils positive for CD63). Boxes represent interquartile range (IQR) with whiskers extending 1.5 the IQR. Lines connect the medians across time. Points represent individual volunteers. Abbreviations: BAT=basophil activation test, BPE=birch pollen extract, Ig=immunoglobin, nMLF= nasal mucosal lining fluid

**Figure S5: Selected allergen-specific serum Ig responses, changes in basophil activation test (BAT) and titrated skin prick test (tSPT) in volunteers with (IL-13R, red, n=8) or without late phase allergic IL-13 response (IL-13NR, yellow, n=12) during birch pollen extract (BPE) provocation vs. those being provoked with placebo (Placebo, grey, n=10).** Boxplots (A-C) display serum log2 fold changes compared to baseline (x-axis) in allergen specific IgE (A), IgG1 (B), IgG4 (C) across time (y-axis, weeks) in indicated groups following nasal challenge. The fitted dose-response curves of each subgroup for the BAT (D) are shown at each time point with points representing individual volunteers (x-axis, concentration of Bet v 1 exposure in ng/ml, y-axis, % of total basophils positive for CD63). (E) Median log change from baseline in endpoint titration to birch extract tSPT (y-axis) in placebo and the birch extract subgroups across time (x-axis). For (A-C, E) boxes represent the interquartile range (IQR) with whiskers extending 1.5 times the IQR. Asterisks represent statistically significant differences between groups (*: p≤0.05, **: p≤0.01, ***: p≤0.001, ****: p≤0.0001). Abbreviations: BAT = basophil activation test, CD=cluster of differentiation, Ig=immunoglobin, tSPT= titrated skin prick test, nMLF= nasal mucosal lining fluid, PBMCs = peripheral blood mononuclear cells.

**Supplementary Figure 6: Changes in PNIF and IL-13 during visit N1 in volunteers with (IL-13R, red, n=8) or without late phase allergic IL-13 responses (IL-13NR, yellow, n=12) after birch pollen extract provocation vs. those being provoked with placebo (Placebo, grey, n=10).** Boxplots display mean fold change compared to baseline (A) (x-axis) or IL-13 concentration in pg/ml (B) (x-axis) in PNIF (A) and IL-13 in nMLF (B) across time (y-axis, weeks) in indicated groups following nasal challenge. Boxes represent the interquartile range (IQR) with whiskers extending 1.5 times the IQR. Asterisks represent statistically significant differences between groups (*: p≤0.05, **: p≤0.01, ***: p≤0.001, ****: p≤0.0001). Abbreviations: IL=interleukin, IQR=inter quartile range, PNIF=peak nasal inspiratory flow.

**Supplementary Figure 7: Changes in Bet v 1 specific IgE, IgG1 and IgG4 in nMLF in volunteers with (IL-13R, red, n=8) or without late phase allergic IL-13 responses (IL-13NR, yellow, n=12) after birch pollen extract provocation vs. those being provoked with placebo (Placebo, grey, n=10).** Boxplots (A-C) display nMLF log2 fold changes compared to baseline (x-axis) in allergen specific IgE (A), IgG1 (B), IgG4 (C) across time (y-axis, weeks) in indicated groups following nasal challenge. Boxes represent the interquartile range (IQR) with whiskers extending 1.5 times the IQR. Asterisks represent statistically significant differences between groups (*: p≤0.05, **: p≤0.01, ***: p≤0.001, ****: p≤0.0001). Abbreviations: Ig=immunoglobin, nMLF= nasal mucosal lining fluid, IQR=inter quartile range.

**Supplementary Figure 8: Selected clinical and immunological responses to seasonal allergen exposure in relation to IL-13 responder status in the seasonal arm of the study. IL-13 responder (IL-13R, red, n=8) vs. IL-non-responder 13 response (IL-13NR, orange, n=12) vs. placebo (Placebo, grey, n=10.** **(A)** Overlay of combined symptom medication scores (right y-axis, score 0-3) and birch pollen concentrations (left y-axis, pollen grains per m^3^ air) against time (x-axis, days). **(B-E)** Log2 fold changes compared to baseline (y-axis) of Bet v 1 specific IgE **(B,C)** and IgG **(D, E)** across time (x-axis, weeks) in serum (**B,D**) and nMLF (**C,E**) measured by ELISA. Boxes represent interquartile ranges (IQR) with whiskers extending 1.5 the IQR. Lines are connecting the medians across time. **(F)** Changes in BAT sensitivity shown via fitted dose-response curves at each time point (x-axis, concentration of Bet v 1 exposure in ng/ml, y-axis, % of total basophils positive for CD63) before and after the birch pollen season. **(G)** Changes in endpoint titration as compared to baseline in the birch extract tSPT. For **(A and G)** points represent the median and for **(B,C,D and E)** points represent individual volunteers. Abbreviations: BAT: basophil activation test, CD: Cluster of differentiation, ELISA: Enzyme-Linked Immunosorbent Assay, Ig=immunoglobin, IL=interleukin, IQR=inter quartile range, nMLF= nasal mucosal lining fluid, tSPT=titrated skin prick test.

**3. References**

1.	Campion NJ, Villazala-Merino S, Thwaites RS, Stanek V, Fd HK, Pertsinidou E, et al. Nasal IL-13 production identifies patients with late phase allergic responses. J Allergy Clin Immunol. 2023.

2.	Heinzerling L, Mari A, Bergmann KC, Bresciani M, Burbach G, Darsow U, et al. The skin prick test - European standards. Clin Transl Allergy. 2013;3(1):3.

3.	Niederberger V, Marth K, Eckl-Dorna J, Focke-Tejkl M, Weber M, Hemmer W, et al. Skin test evaluation of a novel peptide carrier-based vaccine, BM32, in grass pollen-allergic patients. J Allergy Clin Immunol. 2015;136(4):1101-3 e8.

4.	Karsonova A, Riabova K, Villazala-Merino S, Campana R, Niederberger V, Eckl-Dorna J, et al. Highly sensitive ELISA-based assay for quantification of allergen-specific IgE antibody levels. Allergy. 2020;75(10):2668-70.

5.	Flicker S, Steinberger P, Eibensteiner PB, Lebecque S, Kraft D, Valenta R. Molecular characterization of a human immunoglobulin G4 antibody specific for the major birch pollen allergen, Bet v 1. Clin Exp Allergy. 2008;38(2):365-73.

6.	Tulaeva I, Cornelius C, Zieglmayer P, Zieglmayer R, Schmutz R, Lemell P, et al. Quantification, epitope mapping and genotype cross-reactivity of hepatitis B preS-specific antibodies in subjects vaccinated with different dosage regimens of BM32. EBioMedicine. 2020;59:102953.

7.	Pfaar O, Demoly P, Gerth van Wijk R, Bonini S, Bousquet J, Canonica GW, et al. Recommendations for the standardization of clinical outcomes used in allergen immunotherapy trials for allergic rhinoconjunctivitis: an EAACI Position Paper. Allergy. 2014;69(7):854-67.
