# Supplementary material for: Kinetics of Antibody Responses and Effector Cell Sensitivity After High Dose Birch Extract Nasal Challenge
Source: Allergy. 2026 May 4;81(11):3920–35. doi: 10.1111/all.70365 (PMC13753670; doi:10.1111/all.70365)
Supplement: Supplementary file 10 [file ALL-81-3920-s006.docx]

**Table S3: BAT Sensitivity and Reactivity.**

| **Time point** | **Parameter** | **Placebo**^1^ | **Birch Extract**^1^ | ***p*-value**^2^ |
| --- | --- | --- | --- | --- |
| Nasal Challenge Arm | | | | |
| Baseline | ED50 | 0.26 (0.12-0.4) | 0.18 (0.1-0.27) | 0.3545 |
| Baseline | CD63_max_ | 66.51 (58.07-74.95) | 68.65 (61.62-75.68) | 0.7012 |
| 2 Weeks | ED50 | 0.28 (0.09-0.47) | 0.3 (0.17-0.42) | 0.8837 |
| 2 Weeks | CD63_max_ | 58.6 (49.18-68.02) | 59.86 (53.78-65.95) | 0.8242 |
| 4 Weeks | ED50 | 0.4 (0.14-0.66) | 0.17 (0.1-0.24) | 0.0985 |
| 4 Weeks | CD63_max_ | 60.96 (50.2-71.71) | 67 (60.93-73.06) | 0.3362 |
| 6 Weeks | ED50 | 0.24 (0.13-0.36) | 0.18 (0.12-0.24) | 0.3109 |
| 6 Weeks | CD63_max_ | 62.76 (55.65-69.88) | 67.62 (62.51-72.73) | 0.2753 |
| 8 Weeks | ED50 | 0.25 (0.11-0.38) | 0.18 (0.11-0.25) | 0.3758 |
| 8 Weeks | CD63_max_ | 59.91 (52.26-67.55) | 69.42 (63.75-75.1) | 0.0501 |
| 10 Weeks | ED50 | 0.27 (0.14-0.41) | 0.21 (0.14-0.28) | 0.4117 |
| 10 Weeks | CD63_max_ | 57.69 (50.36-65.02) | 70.48 (64.93-76.02) | 0.0066 |
| 12 Weeks | ED50 | 0.2 (0.07-0.33) | 0.17 (0.1-0.24) | 0.6786 |
| 12 Weeks | CD63_max_ | 55.18 (46.97-63.39) | 67.78 (61.78-73.77) | 0.0153 |
| 16 Weeks | ED50 | 0.36 (0.18-0.54) | 0.22 (0.15-0.3) | 0.1615 |
| 16 Weeks | CD63_max_ | 51.36 (44.19-58.52) | 67.23 (61.7-72.75) | 0.0007 |
| Seasonal Arm | | | | |
| Pre Birch Season | ED50 | 0.27 (0.12-0.42) | 0.2 (0.12-0.28) | 0.4175 |
| Pre Birch Season | CD63_max_ | 53.01 (45.92-60.1) | 65.61 (59.67-71.55) | 0.0078 |
| 4 Weeks Post Birch Season | ED50 | 0.29 (0.15-0.44) | 0.25 (0.17-0.34) | 0.6228 |
| 4 Weeks Post Birch Season | CD63_max_ | 52.37 (46.13-58.61) | 69.97 (64.44-75.5) | 4.6x10^-5^ |
| 8 Weeks Post Birch Season | ED50 | 0.23 (0.09-0.38) | 0.17 (0.09-0.26) | 0.4768 |
| 8 Weeks Post Birch Season | CD63_max_ | 58.63 (50.06-67.2) | 64.78 (57.8-71.76) | 0.2741 |
| 20 Weeks Post Birch Season | ED50 | 0.37 (0.11-0.64) | 0.24 (0.1-0.37) | 0.3538 |
| 20 Weeks Post Birch Season | CD63_max_ | 47.73 (37.82-57.64) | 61.19 (53.01-69.36) | 0.0401 |

^1^ estimate (95% confidence interval)

^2^ *t*-test

ED50 = BAT Sensitivity, CD63_max_ = BAT Reactivity
