# Supplementary material for: Kinetics of Antibody Responses and Effector Cell Sensitivity After High Dose Birch Extract Nasal Challenge
Source: Allergy. 2026 May 4;81(11):3920–35. doi: 10.1111/all.70365 (PMC13753670; doi:10.1111/all.70365)
Supplement: Supplementary file 11 [file ALL-81-3920-s007.docx]

**Table S1: Patient Demographics and Sensitization Profile.**

| **Patient ID** | **Group** | **Responder Status** |  | **Sex** | **Age** |  | **Symptomatic Months^∗^** | **Allergic Symptoms^†^** | **Co-sensitisations^‡^** |
| --- | --- | --- | --- | --- | --- | --- | --- | --- | --- |
| 01 | Placebo | NA |  | 29 | F |  | 1-6 | C + R | T, G, W, A |
| 02 | Placebo | NA |  | 40 | M |  | “Birch Season” | C + R | T, W |
| 03 | Placebo | NA |  | 43 | F |  | 2-4 | C + R + AA | G, W, HDM, V |
| 04 | Placebo | NA |  | 27 | F |  | 4 | R | T, G, W, HDM, A |
| 05 | Placebo | NA |  | 24 | F |  | 3-5 | C + R | T, G, W, F |
| 06 | Placebo | NA |  | 25 | F |  | 2-4 | C + R + AA | T, G, W |
| 07 | Placebo | NA |  | 28 | F |  | 4-9 | C + R + AA | T, G, W |
| 08 | Placebo | NA |  | 30 | M |  | 3-4 | C + R | T, G, W |
| 09 | Placebo | NA |  | 24 | F |  | 3-4 | C + R | T, G, W |
| 10 | Placebo | NA |  | 37 | F |  | 3-5 | C + R + AD | T, G, W, HDM, F |
| 11 | Birch Extract | IL-13^R^ |  | 29 | F |  | 4-5 | C + R + AA + AD | T, G, A |
| 12 | Birch Extract | IL-13^NR^ |  | 41 | M |  | 4-8 | C + R | T, G, W, F |
| 13 | Birch Extract | IL-13^R^ |  | 28 | F |  | 2-5 | C + R | T, A |
| 14 | Birch Extract | IL-13^NR^ |  | 47 | F |  | 4-5 | C + R + AA | T, A |
| 15 | Birch Extract | IL-13^NR^ |  | 20 | F |  | 3-6 | C + R | T, G, W, A |
| 16 | Birch Extract | IL-13^NR^ |  | 21 | M |  | 3-5 | C + R | T, G, W, HDM |
| 17 | Birch Extract | IL-13^NR^ |  | 49 | F |  | 3-5 | C + AD | T, G, W, A |
| 18 | Birch Extract | IL-13^R^ |  | 29 | M |  | 4-5 | C + R | T, G, W, HDM |
| 19 | Birch Extract | IL-13^NR^ |  | 25 | M |  | 3-5 | C + R + AA | T, G, W |
| 20 | Birch Extract | IL-13^R^ |  | 25 | F |  | 2-5 | C + R | T, G, W |
| 21 | Birch Extract | IL-13^R^ |  | 25 | F |  | 3-4 | C + R | T |
| 22 | Birch Extract | IL-13^NR^ |  | 28 | F |  | 3-6 | C + R + AA | T, G, W |
| 23 | Birch Extract | IL-13^NR^ |  | 25 | M |  | 3-4 | C + R | T, G, W, HDM |
| 24 | Birch Extract | IL-13^R^ |  | 42 | M |  | 4 | C + R | T, A |
| 25 | Birch Extract | IL-13^R^ |  | 43 | F |  | 3-4 | C + R | T, A |
| 26 | Birch Extract | IL-13^NR^ |  | 25 | F |  | 3-4 | C + R + AA | T, G, W |
| 27 | Birch Extract | IL-13^NR^ |  | 25 | M |  | 3-5 | C + R + AA | T, G, W |
| 28 | Birch Extract | IL-13^NR^ |  | 33 | F |  | 4-7 | C + R | T, G, W, F |
| 29 | Birch Extract | IL-13^R^ |  | 22 | F |  | 4-8 | C + R + AA | T, G, W, A |
| 30 | Birch Extract | IL-13^NR^ |  | 22 | M |  | 4-5 | C + R | T, G, W, A |

IL-13^R^ = IL-13 Responder, IL-13NR = IL-13 Non-responder, F = Female, M = Male, nMLF = Nasal mucosal lining fluid, BAT= Basophil Activation Test, ED50 = Effective dose at 50% activation

∗ The number corresponds to the calendar year months: 1 = January, 2 = February, etc.

† Allergic symptoms: C = conjunctivitis, R = rhinitis, AA = allergic asthma, and AD = atopic dermatitis or pruritus.

‡ Cosensitizations: T = tree pollen, G = grass pollen, W = weed pollen, A = animal dander, HDM = house dust mite, V = venom, and F = fungi.
