# Supplementary material for: Kinetics of Antibody Responses and Effector Cell Sensitivity After High Dose Birch Extract Nasal Challenge
Source: Allergy. 2026 May 4;81(11):3920–35. doi: 10.1111/all.70365 (PMC13753670; doi:10.1111/all.70365)
Supplement: Supplementary file 12 [file ALL-81-3920-s010.docx]

**Table S2: Patient Baseline Antibody and Effector Responses.**

|  |  |  |  | **ELISA** | | | | | | | | | |  | **BAT**  **(Bet v 1 (ng/mL))** |  | **tSPT** |
| --- | --- | --- | --- | --- | --- | --- | --- | --- | --- | --- | --- | --- | --- | --- | --- | --- | --- |
| **Patient ID** | **Group** | **Responder Status** |  | **nMLF sIgE (ng/mL)** | **Serum sIgE (ng/mL)** | **nMLF**  **sIgA (OD)** | **Serum sIgA (OD)** | **nMLF sIgG (OD)** | **Serum sIgG (OD)** | **nMLF sIgG1 (OD)** | **Serum sIgG1 (OD)** | **nMLF sIgG4 (ng/mL)** | **Serum sIgG4 (ng/mL)** |  | **ED50** |  | **SBE/mL** |
| 01 | Placebo | NA |  | 12.16 | 92.87 | 1.79 | 0.34 | 0.28 | 0.45 | 1.68 | 0.28 | 254.89 | 1504.38 |  | 0.25 |  | 69 |
| 02 | Placebo | NA |  | 0.99 | 4.89 | 1.80 | 2.19 | 0.40 | 0.15 | 0.28 | 0.23 | 466.01 | 1565.74 |  | 0.31 |  | 69 |
| 03 | Placebo | NA |  | 4.83 | 89.51 | 0.48 | 0.28 | 0.47 | 1.51 | 1.35 | 2.55 | 255.27 | 4032.59 |  | 0.31 |  | 617 |
| 04 | Placebo | NA |  | 2.03 | 12.75 | 0.67 | 0.83 | 1.74 | 0.84 | 3.08 | 0.77 | 713.64 | 9198.06 |  | 0.52 |  | 617 |
| 05 | Placebo | NA |  | 13.21 | 53.12 | 0.72 | 0.94 | 0.20 | 1.00 | 0.77 | 2.07 | 71.79 | 591.54 |  | 0.06 |  | 69 |
| 06 | Placebo | NA |  | 1.15 | 88.94 | 0.27 | 0.31 | 0.39 | 0.49 | 0.02 | 0.65 | 24.29 | 631.24 |  | 0.26 |  | 206 |
| 07 | Placebo | NA |  | 0.78 | 19.54 | 0.24 | 0.13 | 0.54 | 0.52 | 0.02 | 0.24 | 45.60 | 837.63 |  | 0.99 |  | 617 |
| 08 | Placebo | NA |  | 77.77 | 128.32 | 1.27 | 0.10 | 0.42 | 0.33 | 0.41 | 0.25 | 415.33 | 1344.41 |  | 0.05 |  | 206 |
| 09 | Placebo | NA |  | 19.76 | 120.19 | 2.78 | 0.23 | 1.87 | 1.81 | 3.02 | 1.83 | 156.88 | 3616.13 |  | 0.67 |  | 206 |
| 10 | Placebo | NA |  | 11.23 | 65.25 | 0.79 | 0.49 | 0.89 | 1.08 | 1.00 | 0.73 | 992.32 | 16959.69 |  | 0.47 |  | 617 |
| 12 | Birch Extract | IL-13^NR^ |  | 0.85 | 32.89 | 0.76 | 0.83 | 0.16 | 0.84 | 0.15 | 0.07 | 254.36 | 26801.27 |  | 0.27 |  | 617 |
| 14 | Birch Extract | IL-13^NR^ |  | 8.88 | 88.25 | 0.69 | 0.45 | 0.16 | 0.49 | 0.08 | 0.70 | 70.27 | 4513.49 |  | 0.08 |  | 617 |
| 15 | Birch Extract | IL-13^NR^ |  | 10.62 | 48.66 | 0.71 | 0.74 | 1.13 | 1.30 | 0.16 | 0.29 | 187.26 | 3086.67 |  | 0.18 |  | 617 |
| 16 | Birch Extract | IL-13^NR^ |  | 6.26 | 18.98 | 0.74 | 0.31 | 0.29 | 0.37 | 2.07 | 0.58 | 135.16 | 1216.60 |  | 0.10 |  | 617 |
| 17 | Birch Extract | IL-13^NR^ |  | 2.67 | 17.92 | 0.22 | 0.96 | 0.39 | 0.36 | 0.80 | 0.17 | 620.32 | 3628.65 |  | 0.43 |  | 206 |
| 19 | Birch Extract | IL-13^NR^ |  | 1.11 | 34.45 | 2.99 | 0.57 | 1.19 | 1.70 | 0.56 | 0.89 | 667.27 | 22079.85 |  | 1.07 |  | 617 |
| 22 | Birch Extract | IL-13^NR^ |  | 2.63 | 150.73 | 0.71 | 0.46 | 0.30 | 0.46 | 0.01 | 0.16 | 170.46 | 11581.46 |  | 0.06 |  | 617 |
| 23 | Birch Extract | IL-13^NR^ |  | 8.75 | 914.01 | 0.21 | 0.34 | 0.06 | 0.36 | 0.01 | 0.10 | 37.23 | 6937.78 |  | 0.05 |  | 617 |
| 26 | Birch Extract | IL-13^NR^ |  | 1.31 | 7.78 | 2.80 | 0.21 | 0.70 | 0.74 | 0.13 | 0.27 | 208.76 | 11214.02 |  | 0.52 |  | 5556 |
| 27 | Birch Extract | IL-13^NR^ |  | 3.19 | 38.77 | 0.15 | 1.48 | 0.09 | 1.15 | 0.06 | 1.14 | 20.31 | 554.52 |  | 0.68 |  | 206 |
| 28 | Birch Extract | IL-13^NR^ |  | 0.78 | 12.77 | 0.13 | 0.20 | 0.42 | 1.33 | 0.06 | 2.42 | 52.90 | 4694.96 |  | 1.03 |  | 1852 |
| 30 | Birch Extract | IL-13^NR^ |  | 16.73 | 713.63 | 0.39 | 0.53 | 0.07 | 0.85 | 0.03 | 1.34 | 8.93 | 3263.28 |  | 0.07 |  | 617 |
| 11 | Birch Extract | IL-13^R^ |  | 3.90 | 10.50 | 0.25 | 0.92 | 0.06 | 0.61 | 0.12 | 0.67 | 179.18 | 7025.75 |  | 0.11 |  | 206 |
| 13 | Birch Extract | IL-13^R^ |  | 3.68 | 9.57 | 0.61 | 0.49 | 0.52 | 0.13 | 0.10 | 0.15 | 116.13 | 806.36 |  | 0.04 |  | 617 |
| 18 | Birch Extract | IL-13^R^ |  | 7.56 | 1210.55 | 1.20 | 0.44 | 0.34 | 1.17 | 0.30 | 0.88 | 286.54 | 14633.97 |  | 0.76 |  | 206 |
| 20 | Birch Extract | IL-13^R^ |  | 20.27 | 38.84 | 0.25 | 0.15 | 1.04 | 0.59 | 0.20 | 0.26 | 377.62 | 16046.31 |  | 0.26 |  | 617 |
| 21 | Birch Extract | IL-13^R^ |  | 5.05 | 611.74 | 0.59 | 0.18 | 0.18 | 0.90 | 0.03 | 0.60 | 92.32 | 21506.35 |  | 0.07 |  | 23 |
| 24 | Birch Extract | IL-13^R^ |  | 6.40 | 27.26 | 0.43 | 0.23 | 0.16 | 0.28 | 0.08 | 0.25 | 50.13 | 316.80 |  | 0.05 |  | 69 |
| 25 | Birch Extract | IL-13^R^ |  | 4.18 | 10.63 | 0.66 | 0.25 | 0.86 | 0.33 | 0.60 | 0.11 | 362.56 | 1914.63 |  | 0.32 |  | 206 |
| 29 | Birch Extract | IL-13^R^ |  | 4.18 | 693.17 | 0.44 | 1.05 | 0.12 | 0.84 | 0.12 | 1.07 | 53.79 | 11871.38 |  | 0.64 |  | 617 |

IL-13^R^ = IL-13 Responder; IL-13^NR^ = IL-13 Non-responder; ELISA = Enzyme-linked immunosorbent assay; BAT = Basophil activation test; tSPt = Titrated skin prick test; OD = Optical density; ED50 = Effective dose at 50% activation of CD63+ basophiles; SBE/mL = Standardized Biological Units per milliliter; nMLF = Nasal mucosal lining fluid. ELISA values represent the mean of triplicates after background signals were accounted for. For IgE and IgG4 standards were available and therefore values are reported in ng/ml, for IgA, IgG and IgG1 no standards were available and therefore mean OD is reported. SBE/ml represents the units of allergen concentration as provided by manufacturer of the birch extract (Bencard Allergie, Munich, Germany).
